# Phylogenetic reconstruction of Syntermitinae (Isoptera, Termitidae) based on morphological and molecular data

**DOI:** 10.1371/journal.pone.0174366

**Published:** 2017-03-22

**Authors:** Mauricio M. Rocha, Adriana C. Morales-Corrêa e Castro, Carolina Cuezzo, Eliana M. Cancello

**Affiliations:** 1 Museu de Zoologia da Universidade de São Paulo, São Paulo, SP, Brazil; 2 Departamento de Biologia Aplicada à Agropecuária, FCAV-UNESP Campus de Jaboticabal, SP, Brazil; Museum National d'Histoire Naturelle, FRANCE

## Abstract

The subfamily Syntermitinae comprises a group of Neotropical termites with 18 genera and 101 species described. It has been considered a natural group, but relationships among the genera within the subfamily remain uncertain, and some genera appear to be non-monophyletic. Here, we provide a comprehensive phylogeny including six Neotropical species of Termitinae as outgroup, 42 Syntermitinae species as ingroup, 92 morphological characters (from external and internal anatomy of soldier and worker castes) and 117 molecular sequences (109 obtained for this study and 8 from GenBank) of 4 gene regions (41 and 22 from Cytochrome Oxidase I and II respectively, 19 from Cytochrome b, and 35 from 16S rDNA). Morphological and molecular data were analyzed in combination, with the Bayesian inference method, and the important aspects of termite biology, defense and feeding habits are discussed based on the resulting tree. Although useful for providing diagnostic characters, the morphology of the soldier caste reveals several cases of convergence; whereas the feeding habit shows indications of evolutionary significance.

## Introduction

The subfamily Syntermitinae comprises a group of Neotropical termites that ranges from southern Mexico (*Cahuallitermes*) to northern Argentina (*Cornitermes*, *Procornitermes*, *Rhynchotermes*, *Syntermes*), with the richest generic and specific diversity in the Brazilian Cerrado biome. Fifteen syntermitine genera occur in the Cerrado, where several species of *Cornitermes*, *Silvestritermes* and *Syntermes* construct conspicuous epigeal nests that characterize this savanna-like landscape. *Cornitermes cumulans* can reach a nest density of 55/ha, and is considered a keystone species in the Cerrado [1]. These termite nests may harbor many other termite species as well as other groups of invertebrates.

The feeding and nesting habits of syntermitine species are diverse. The group includes grass/litter-feeders, intermediate feeders, and humus-feeders. The nests are variable; some species build earthen nests; most are commonly epigeal, but arboreal and subterranean forms are well known. Other nesting habits include inquilines, reformers, and diffuse galleries in the ground.

A total of 18 genera and 101 species are now established as part of the subfamily. Some of the taxa treated in taxonomic revisions and original descriptions in the last 20 years are *Acangaobitermes* [2], *Armitermes* [3], *Cahuallitermes* [4], *Cyrilliotermes* [5], *Curvitermes* [6], *Labiotermes* [7], *Macuxitermes* [8, 9], *Noirotitermes* [10], *Paracurvitermes* [11], *Rhynchotermes* [12], and *Syntermes* [13]. However, the status of *Embiratermes* is still in need of revision [3].

Engel and Krishna [14] proposed the subfamily, including only *Cornitermes*, *Labiotermes*, *Procornitermes* and *Syntermes*. Lately, Constantino and Carvalho [11] gave a new diagnosis, considering all the genera of “mandibulate nasutes” then described (those genera with soldiers having developed mandibles and a recognizable frontal tube). Although Syntermitinae is a recently proposed taxonomic category, the “mandibulate nasutes” group was recognized very early in the termite literature. The “mandibulate nasutes” together with “true nasutes” (group of genera with soldiers having vestigial mandibles and a developed frontal tube) were considered part of the worldwide subfamily Nasutitermitinae.

In the last century, two hypotheses were proposed regarding the origin of the nasute soldier: the monophyletic hypothesis, where the “mandibulate soldiers” form an ancestral group of the “true nasutes” [15, 16]; and the diphyletic hypothesis, where two independent lineages of “mandibulate soldiers” led to the “true nasutes” [17–20].

Inward and collaborators [21], in a comprehensive phylogenetic analysis with morphological and molecular data, supported the hypothesis that “mandibulate nasutes” and “true nasutes” are two distinct, independent lineages, and that the Syntermitinae is more closely related to the Amitermes-group (Termitinae) than to the Nasutitermitinae.

Rocha and collaborators [3] developed a revisionary proposal for the genus *Armitermes “sensu lato”*, which included a cladistic analysis involving morphological characters from all species of *Armitermes* and representatives of all genera of “mandibulate nasutes”. In this phylogenetic approach, the genus *Armitermes* appears as polyphyletic, and some species are relocated to new genera, although the relationships among Syntermitinae genera are poorly resolved.

Herein, we propose a comprehensive phylogenetic hypothesis for Syntermitinae, based on combined morphological and molecular data under a Bayesian approach; and reconstruct some aspects of the defense behavior and feeding habits of the group.

## Material and methods

### Taxon sampling and outgroup selection

We included a total of 42 syntermitine species as ingroup, representing the diversity of the 18 currently described syntermitine genera; and 6 species of Termitinae as outgroup, chosen for their established relationships to Syntermitinae [21–23] and also based on our experience with Neotropical termites. Morphological studies were carried out on termite specimens deposited in the Isoptera collection of the Museu de Zoologia da Universidade de São Paulo, São Paulo, Brazil (MZUSP). A representative sample of each lot used to perform the molecular studies was formally deposited in the MZUSP as well and appropriately registered for public consult.

### Morphological characters

We included a total of 92 characters, 40 of the soldier external morphology, 42 of the coiling gut *in situ* and the configuration of the different parts of the worker digestive tube, and 10 of worker external morphology. The morphological character data are expanded from our previous study [3]; most characters are referenced in Figs 1–17.

**Fig 1:**
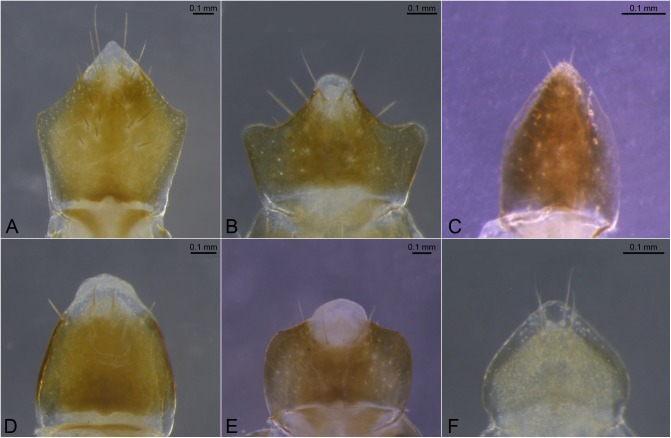
Examples of shapes of labrum. A. *Syntermes molestus*; B. *Procornitermes triacifer*; C. *Microcerotermes strunckii*; D. *Labiotermes labralis*; E. *Cornitermes cumulans* F. *Silvestritermes holmgreni*.

**Fig 2:**
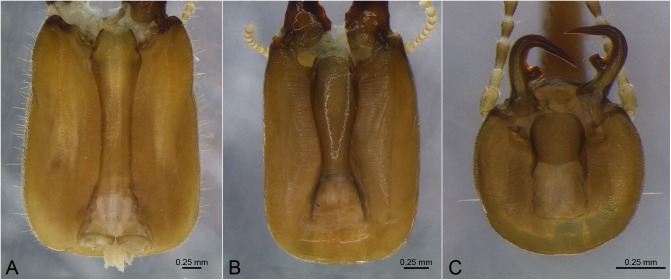
Examples of shapes of postmentum. A. *Cornitermes cumulans*; B. *Labiotermes labralis*; C. *Rhynchotermes nasutissimus*.

**Fig 3:**
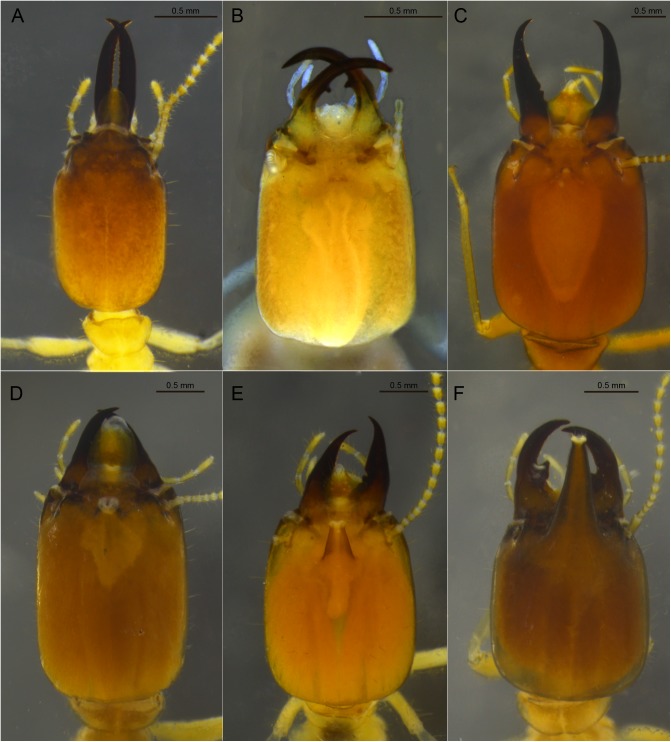
Examples of types of frontal gland openings and frontal tube shapes. A. *Microcerotermes strunckii*; B. *Amitermes amifer*; C. *Syntermes molestus*; D. *Labiotermes labralis*; E. *Procornitermes araujoi*; F. *Embiratermes festivellus*.

**Fig 4:**
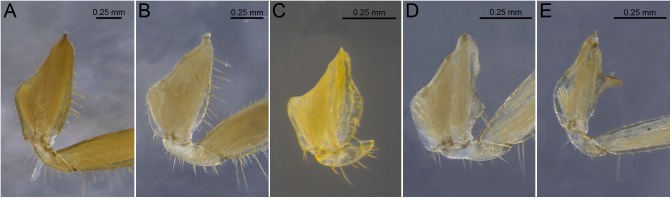
Examples of shapes of outer margins of the forecoxae and projections. A. *Syntermes molestus*; B. *Cornitermes cumulans*; C. *Armitermes spininotus*; D. *Embiratermes festivellus*; E. *Rhynchotermes nasutissimus*.

**Fig 5:**
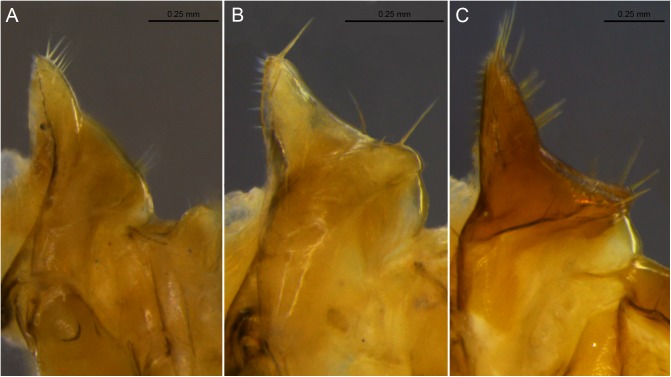
Examples of lateral lobes of the pronotum. A. *Labiotermes labralis*; B. *Embiratermes festivellus*; C. *Syntermes molestus*.

**Fig 6:**
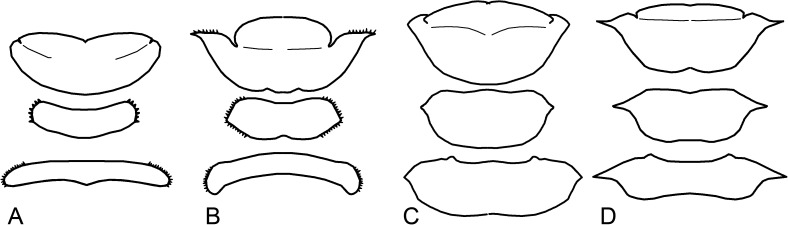
Examples of shapes of thoracic nota. A. *Labiotermes labralis*; B. *Armitermes spininotus*; C. *Syntermes molestus*; D. *Syntermes crassilabrum*.

**Fig 7:**
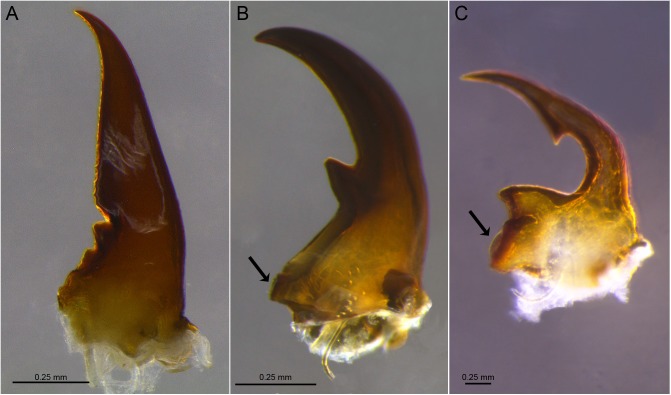
Examples of mandibles (right) and molar regions. A. *Procornitermes araujoi*; B. *Embiratermes festivellus*; C. *Curvitermes odontognathus*. (Arrows indicate the molar region).

**Fig 8:**
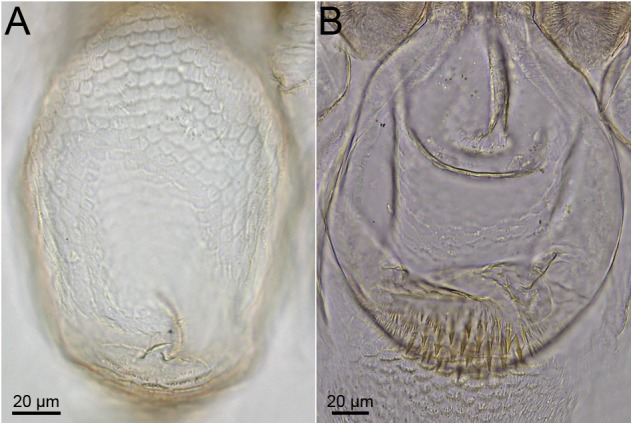
Examples of pulvilli ornamentations. A. *Uncitermes teevani*; B. *Mapinguaritermes peruanus*.

**Fig 9:**
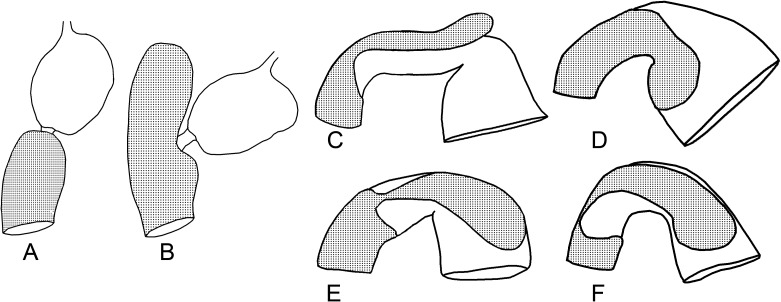
**Insertion of the stomodeal valve in the mesenteron (A, B) and examples of alignment of the mesenteric tongues (C–F)**. A. *Cornitermes cumulans*; B. *Procornitermes striatus*; C. *Silvestritermes holmgreni*; D. *Mapinguaritermes peruanus*; E. *Rhynchotermes nasutissimus*; F. *Ibitermes curupira*.

**Fig 10:**
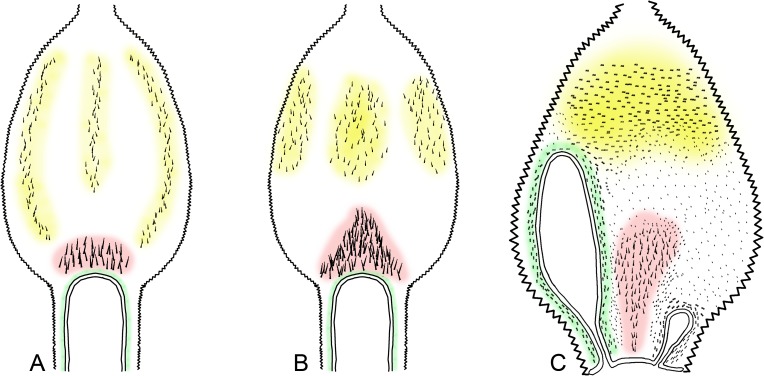
Homology between the ornament regions inside the first proctodeal segment (red, central area; yellow, distal area; and green, region around the mesenteric tongue). A *Curvitermes odontognathus*; B. *Embiratermes festivellus*; C. *Cornitermes cumulans*.

**Fig 11:**
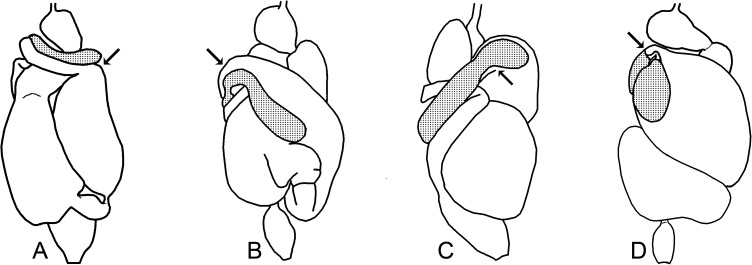
Examples of transition from tubular portion to dilated portion in P1 (arrows, initial portion of the dilated regions). A. *Silvestritermes holmgreni*; B. *Cornitermes cumulans*; C. *Cyrilliotermes angulariceps; D*. *Uncitermes teevani*.

**Fig 12:**
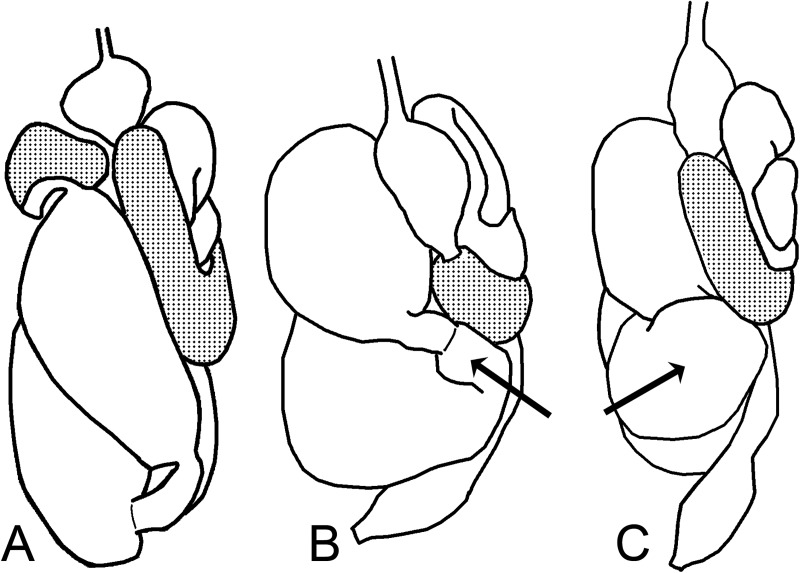
Examples of connections between P1 and P3 through P2. A. *Silvestritermes holmgreni*; B. *Cyrilliotermes angulariceps*; C. *Embiratermes festivellus*.

**Fig 13:**
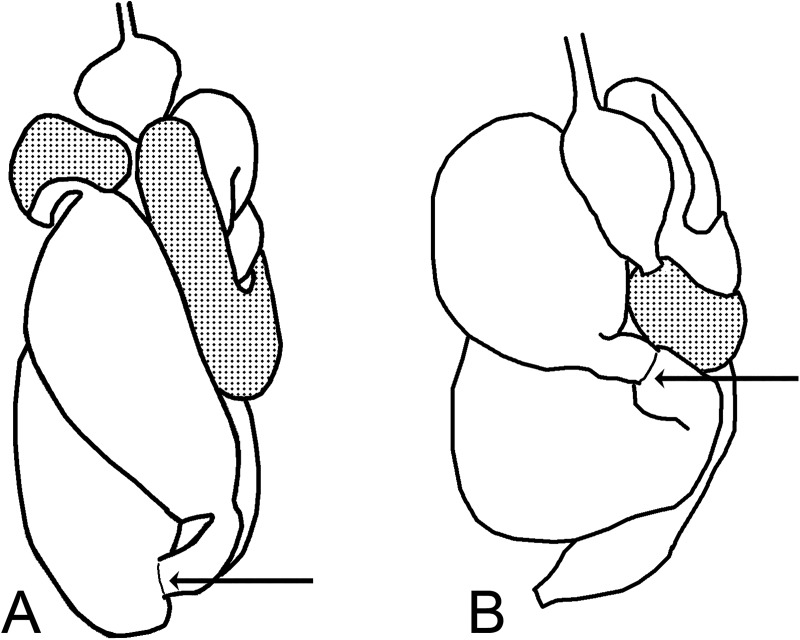
Examples of P2 insertion, relative to abdomen length (arrows, P2 position). A. *Silvestritermes holmgreni*; B. *Cyrilliotermes angulariceps*.

**Fig 14:**
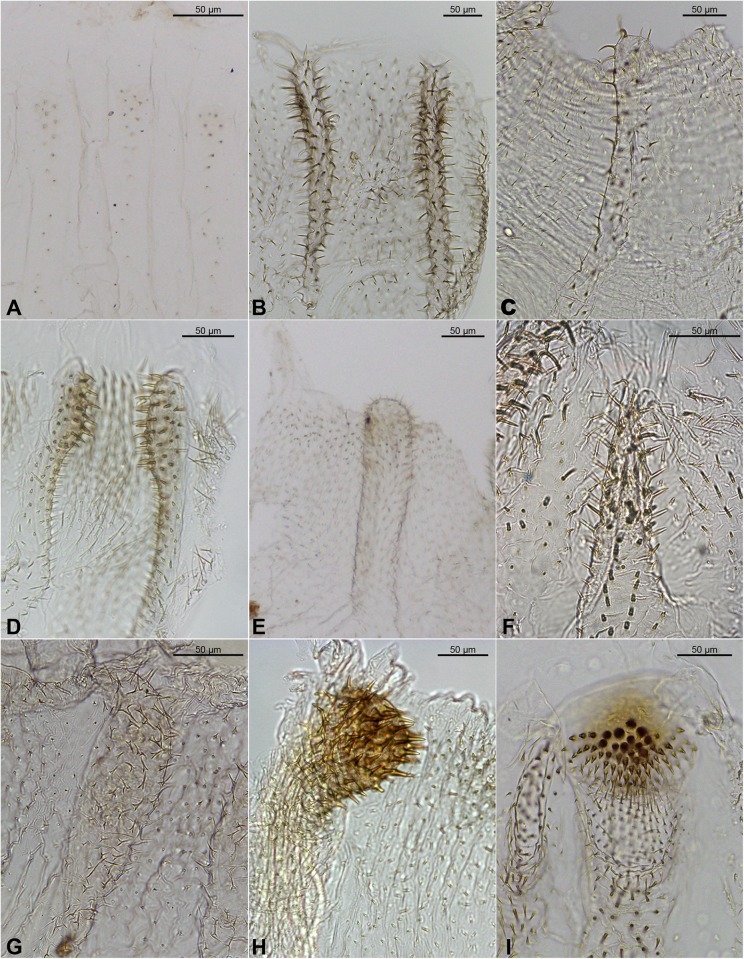
Examples of enteric valve shapes. A. *Amitermes amifer*; B. *Curvitermes odontognathus*; C. *Mapinguaritermes peruanus*; D. *Genuotermes spinifer*; E. *Embiratermes festivellus*; F. *Embiratermes silvestrii*; G. *Procornitermes lespessi*; H. *Cornitermes cumulans*; I. *Silvestritermes holmgreni*.

**Fig 15:**
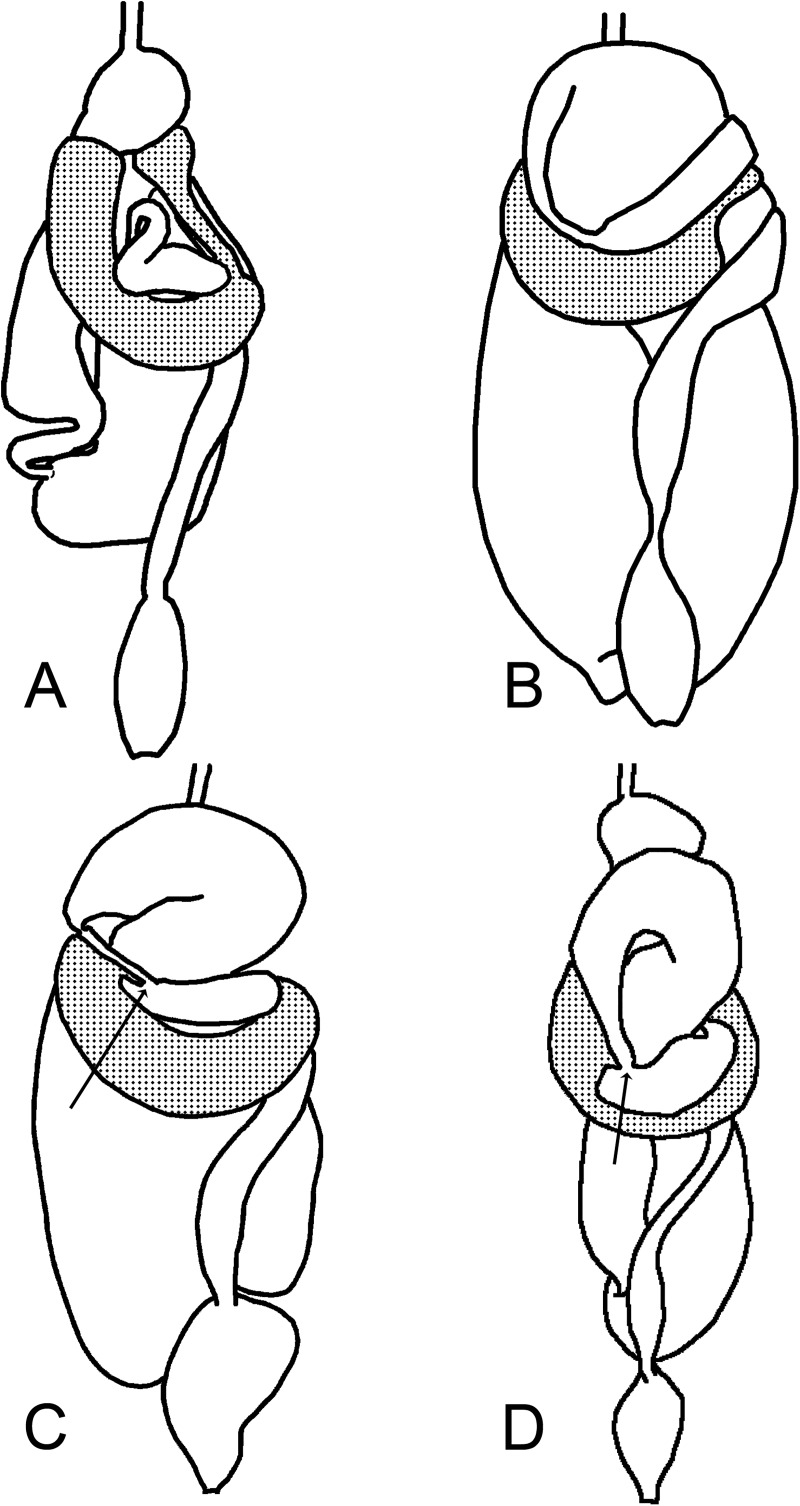
Examples of P3b shapes and isthmus insertions (arrow, sub-apical P3 insertion). A. *Microcerotermes strunckii*; B. *Curvitermes odontognathus*; C. *Embiratermes ignotus*; D. *Acangaobitermes krishnai*.

**Fig 16:**
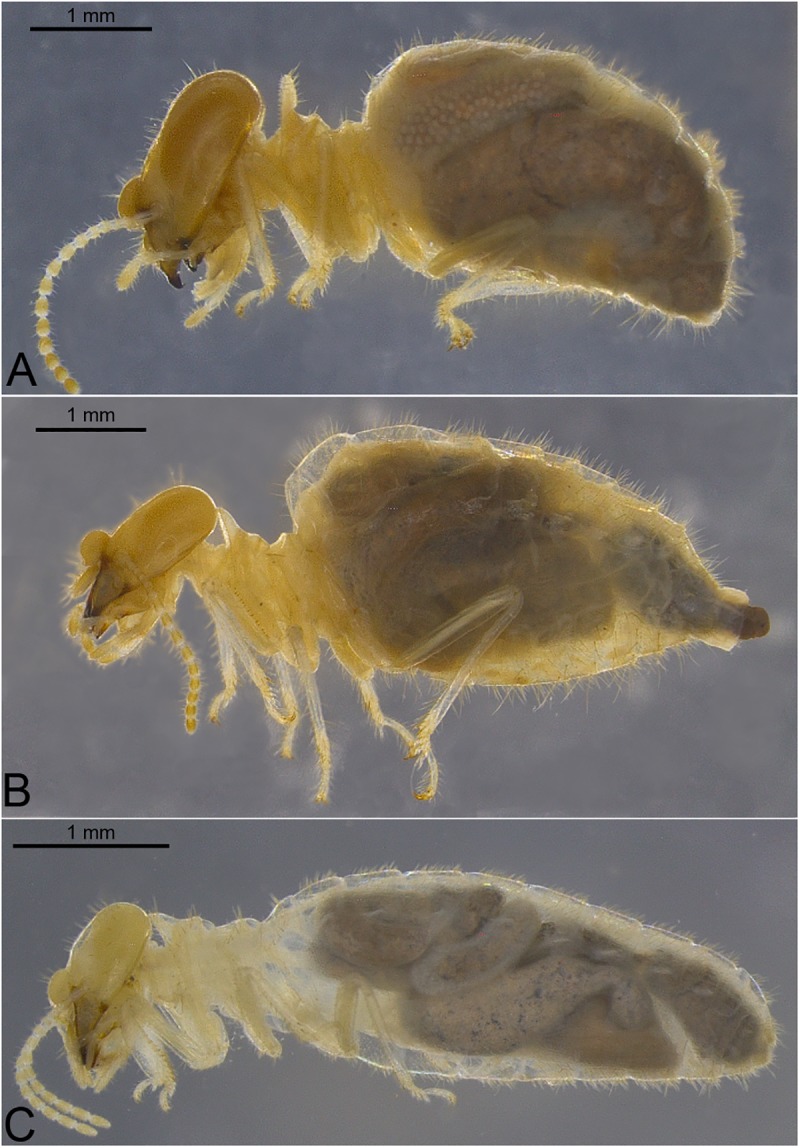
Examples of body proportions and profiles. A. *Cornitermes cumulans*; B. *Labiotermes labralis*; C. *Acangaobitermes krishnai*.

**Fig 17:**
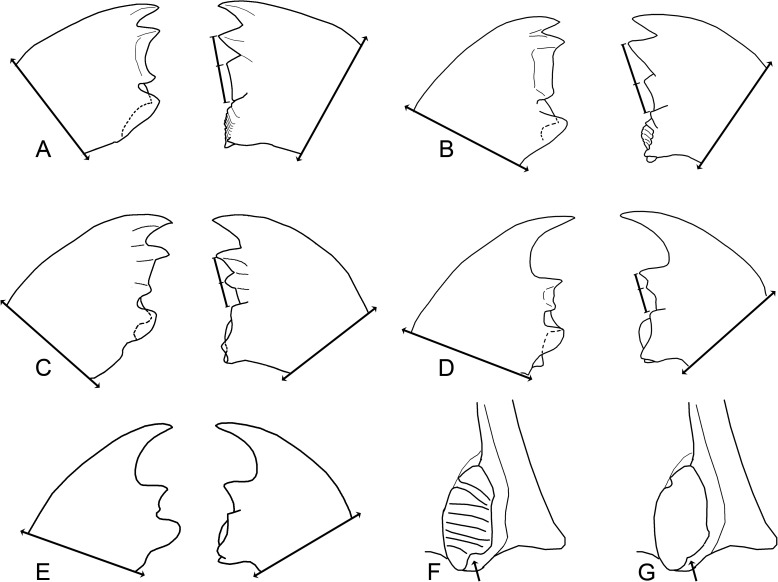
Worker mandibles (not to same scale). A. *Microcerotermes strunckii*; B. *Cornitermes cumulans*; C. *Silvestritermes holmgreni*; D. *Curvitermes odontognathus*; E. *Paracurvitermes manni*; F. detail of *C*. *cumulans* molar plate notch; G. detail of *S*. *holmgreni* molar plate notch.

The character matrix (S1 Table) was edited and managed with Mesquite v.3.04 [24].

#### Soldier head

01. Labrum, hyaline tip: (0) absent (Fig 1C and 1F); (1) present (Fig 1A, 1B, 1D and 1E).

02. Shape of hyaline tip: (0) flat (Fig 1A and 1D); (1) fingerlike (Fig 1B and 1E).

03. Silhouette in dorsal view: (0) cuspidate (Fig 1A, 1B and 1E); (1) lanceolate (Fig 1C); (2) obtuse (Fig 1D and 1F).

04. Cuspidate margins: (0) slender (Fig 1A and 1E); (1) clearly angulate (Fig 1B).

05. Postmentum lateral margins: (0) angled (Fig 2A); (1) sinusoidal (Fig 2B); (2) convex (Fig 2C).

06. Postmentum length: (0) elongated (Fig 2A and 2B); (1) shorter (Fig 2C).

07. Shape of head in dorsal view: (0) rectangular, elongated; (1) rectangular, sort; (2) rounded.

08. Number of antennal articles: (0) 20; (1) 19; (2) 18; (3) 17; (4) 16; (5) 15; (6) 14; (7); 13; (8); 12 (9) 11.

09. Head capsule microsculpture: (0) absent; (1) present (character 7 of [3]).

10. Visibility of frontal pore aperture: (0) indistinct (Fig 3A); (1) distinct (Fig 3B–3F).

11. Frontal pore shape: (0) retracted and narrow (Fig 3B); (1) protruded and wide (Fig 3C–3F).

12. Membranous tissue at frontal pore aperture: (0) absent (Fig 3B and 3C); (1) present (Fig 3D–3F).

13. Frontal projection: (0) absent (Fig 3A–3C); (1) present (Fig 3D and 3E).

14. Length of frontal tube: (0) reaching clypeus (Fig 3E); (1) surpassing clypeus (Fig 3F); (2) lump in profile (Fig 3D); (3) salience (Fig 3C).

#### Soldier thorax

15. Row of stout bristles on outer margins of forecoxae: (0) absent; (1) present (character 29 of [3]).

16. Stout bristles along femur: (0) absent; (1) present (as described for *Labiotermes* [7]).

17. Ornaments on internal face of tibia: (0) flat; (1) row of 20 spines.

18. Tibial spurs formula: (0) 3:2:2; (1) 2:2:2

19. Shape of outer margin of forecoxae: (0) nearly straight (Fig 4A); (1) with lump (Fig 4B).

20. Outer margin of forecoxae, distal portion: (0) round (Fig 4A and 4B); (1) keeled (Fig 4C); (2) spiniform (Fig 4D).

21. Spine on proximal portion of coxae: (0) absent (Fig 4A–4D); (1) present (Fig 4E).

22. Lateral lobes of pronotum: (0) not projected (Fig 5A); (1) slightly projected (Fig 5B); (2) well projected (Fig 5C).

23. Lateral margins of lobes (pronotum): (0) rounded (Fig 6A); (1) angulate (Fig 6C); (2) acuminate (Fig 6B and 6D).

24. Lateral margins of lobes (mesonotum): (0) rounded (Fig 6A); (1) angulate (Fig 6B and 6C); (2) acuminate (Fig 6D).

25. Lateral margins of lobes (metanotum): (0) rounded (Fig 6A and 6B); (1) angulate (Fig 6C); (2) acuminate (Fig 6D).

26. Outer margin of pronotum: (0) smooth (Fig 6A, 6C and 6D); (1) serrated (Fig 6B).

27. Outline of margins of metanotum and mesonotum: (0) smooth (Fig 6C and 6D); (1) serrated (Fig 6A and 6B).

#### Soldier mandibles

28. Molar region: (0) indistinct (Fig 7A); (1) distinct (Fig 7B and 7C).

29. When distinct, relative size of molar region: (0) reduced (Fig 7B); (1) developed (Fig 7C).

30. First marginal tooth of left mandible: (0) absent; (1) present

31. Second marginal tooth of left mandible: (0) absent; (1) present.

32. Shape between first and second marginal teeth of left mandible: (0) “V” concavity; (1) cutting edge

33. Shape of cutting edge between first and second marginal teeth of left mandible: (0) smooth; (1) serrated.

34. First marginal tooth of right mandible: (0) absent; (1) present

35. Second marginal tooth of right mandible: (0) absent; (1) present.

36. Position of second marginal tooth: (0) in proximal portion; (1) in middle.

37. Shape of apical tooth: (0) slender; (1) strong; (2) sinuous.

38. Apical tooth curvature: (0) strongly arched; (1) slightly arched.

39. Internal outline of apical tooth: (0) concave (Fig 7B); (1) sinusoidal (Fig 7A).

40. Subapical tooth: (0) present; (1) absent.

#### Characters of the gut anatomy

41. Gizzard: Ornamentation of first-order pulvilli: (0) without notable ornaments (Fig 8A); (1) with developed spines (Fig 8B).

42. Insertion of stomodeal valve in mesenteron: (0) apical (Fig 9A); (1) subapical (Fig 9B).

43. Mesenteric tongue: (0) absent; (1) present.

44. Mesenteric tongue, proximal portion: (0) robust (Fig 9C, 9D and 9F); (1) constricted (Figs 9E and 11B); (2) filiform.

45. Alignment of mesenteric tongue: (0) continuous with external face of the mesenteric arch (Fig 9C); (1) with apex turned (Fig 9D); (2) laterally to mesenteric arch (Fig 9); (3) twisted (Fig 9F).

46. Secondary mesenteric tongue: (0) absent; (1) present.

47. Malpighian tubules attachment: (0) in two pairs; (1) four united.

48. Large ampulla at insertions of Malpighian tubules: (0) absent; (1) present.

49. Position of Malpighian tubules: (0) internal to mesenteric arch; (1) external to mesenteric arch.

#### Internal ornamentation of first proctodeal segment

The P1 internal ornaments were described by Rocha and Constantini [25], and the homology between the regions adopted in this study is explained in Fig 10 [central area in red, distal area in yellow, and around the mesenteric tongue(s) in green].

50. Ornaments: (0) absent; (1) present.

51. Type of spines covering central area (0) Small spines in rows; (1) aciculiform; (2) robust spines; (3) as thin setae; (4) trifurcated spines.

52. Degree of sclerotization of spines: (0) slightly sclerotized; (1) strongly sclerotized.

53. Pattern of spine coverage in central area: (0) transverse; (1) longitudinal; (2) spaced.

54. Position of spines of central area, relative to mesenteric tongue: (0) after mesenteric tongue; (1) lateral to mesenteric tongue.

55. Central ridges: (0) absent; (1) present.

56. Spines around mesenteric tongue: (0) absent; (1) present.

57. Type of spines around mesenteric tongue: (0) single; (1) in small rows.

58. Coverage in distal area: (0) absent; (1) present.

59. Pattern of coverage in distal area: (0) sparse; (1) grouped in three areas.

60. When grouped in three areas: (0) complete columns; (1) incomplete columns; (2) rounded areas.

#### First proctodeal segment

61. Shape of P1: (0) tubular; (1) dilated.

62. Length of P1 relative to abdomen: (0) nearly the same (relatively outstretched in the abdomen); (1) longer (relatively coiled inside the abdomen).

63. Shape of dilated portion: (0) fusiform; (1) globose.

64. Transition from tubular portion to dilated portion of P1, see arrows: (0) distally constricted (Fig 11A); (1) gradual (Fig 11B); (2) proximally constricted (Fig 11C); (3) very strangulated (Fig 11D).

65. P1 orientation in relation to body axis: (0) parallel (Fig 11A); (1) diagonal (Fig 11B).

66. Shape of P1 final portion: (0) tubular (Fig 12A); (1) conical (Fig 12C); (2) tubular and narrow.

67. Shape of tubular P1 final portion: (0) arched; (1) straight.

#### Second proctodeal segment

68. Position of P2 insertion relative to abdomen length: (0) distally (Fig 13A); (1) at midlength (Fig 13B).

69. Position of P2 insertion in dorsal view: (0) on left side of body; (1) on right side of body.

70. Symmetry of enteric valve armature: (0) hexa-radial; (1) tri-radial; (2) asymmetric.

71. Category of ridges: (0) large pads (Fig 14A); (1) simple ridge (Fig 14B); (2) ridge slightly dilated at apex (Fig 14C); (3) finger-like (Fig 14D–14F); (4) lobate (Fig 14G and 14H); (5) bulbous (Fig 14I).

72. Type of finger-like ridge: (0) protruded (Fig 14D); (1) oblong (Fig 14E); (2) conical (Fig 14F).

73. Type of lobate ridge: (0) slightly lobed (Fig 14G); (1) auricular (Fig 14H).

74. Proportions of lobate ridge: (0) equal; (1) unequal.

75. When slightly dilated at apex, length: (0) elongated; (1) short.

76. Ornaments: (0) absent; (1) present.

77. Ornament coverage: (0) triangular; (1) aciculiform.

#### Third proctodeal segment

78. Initial portion of P3: (0) directly connected (Fig 12); (1) bottleneck (Fig 12B); (2) well-developed enteric valve seating (Fig 12C).

79. Smooth diverticulum of P3a: (0) absent; (1) present.

80. P3b shape in dorsal view: (0) globose (Fig 15B); (1) arched (Fig 15C and 15D); (2) not protruded (Fig 15A).

81. Direction of P3b when arched: (0) turned forward (Fig 15D); (1) turned to right side of body (Fig 15C).

82. Insertion of isthmus: (0) apical (Fig 15A and 15B); (1) sub-apical (Fig 15C and 15D, arrow).

#### Characters based on external morphology of workers

83. Size proportion of head to thorax: (0) head much larger than thorax (Fig 16A); (1) head size similar to thorax (Fig 16B and 16C).

84. Body in profile: (0) slender (Fig 16C); (1) waisted (Fig 16A and 16B).

85. Mandibles: Relative size of left apical tooth: (0) smaller than M1 (Fig 17B); (1) equal to M1 (Fig 17A and 17C); (2) more prominent than M1 (Fig 17D and 17E).

86. Edge of apical tooth: (0) straight (Fig 17A, 17B and 17C); (1) concave (Fig 17D and 17E).

87. M3 tooth on left mandible: (0) present, conspicuous (Fig 17A); (1) present, reduced (Fig 17B–17E).

88. M2 tooth on right mandible: (0) present, conspicuous (Fig 17A–17C); (1) present, reduced (Fig 17D); (2) absent (Fig 17E).

89. Relative position of right M2 tooth: (0) near middle of M1 and molar plate (Fig 17C); (1) near M1 tooth (Fig 17A and 17D); (2) near molar plate (Fig 17B); (3) fused to M1 (Fig 17E).

90. Right M2 posterior margin: (0) straight; (1) concave.

91. Molar plate notch: (0) absent; (1) present, 90 degrees (Fig 17F, arrow); (2) present, more than 90 degrees (Fig 17G, arrow).

92. Molar region: (0) with ridges; (1) with reduced ridges; (2) without ridges.

### Molecular protocols

We chose four regions of the mitochondrial genome, Cytochrome Oxidase I and II (COI ~600 bp, COII ~660 bp), Cytochrome b (Cyt B ~340 bp) and 16S rDNA (~430 bp). The DNA was extracted preferentially from the head and thorax of a single soldier individual preserved in 95% ethanol (Table 1), with the set of reagents from the DNeasy Blood & Tissue Kits (Qiagen), supplemented with 20 mg/ml proteinase K, following the manufacturer’s protocol. The homogenates were incubated at 55°C for 3 h. The gene fragments were then amplified by polymerase chain reaction, PCR [26]. The primers and the amplification conditions are listed in Table 2. PCR was performed in 25 μL reactions (12.5 μL PCR master mix Promega®, 0.6 μM of each primer, 3.0 μL of total DNA, and 3.5 μL deionized water). The amplified PCR products were determined by gel electrophoresis on a 1% agarose gel diluted in TAE buffer (1X) (0.04 M Tris base, 0.02 M acetic acid, and 1 mM EDTA). This same buffer was also used in 1-h electrophoresis runs in an 8-V/cm length gel. All reaction products were purified with Wizard® SV Gel and PCR Clean-Up System (Promega), following the manufacturer’s protocols. Purified PCR products were sequenced with the same primers used in the original PCR reactions and the BigDye® Terminator v3.1 Cycle Sequencing Kit, under the same conditions of PCR. The sense and antisense sequences obtained from each amplicon were assembled, and a consensus sequence for each gene was generated with Geneious v.8.1.7 analysis tools [27]. The nucleotide sequences reported here were submitted to the GenBank database under the accession numbers indicated in Table 1.

**Table 1:** List of GenBank accession codes for each gene.

	MZUSPLot no.	COII	COI	Cytb	16S rDNA
Termitinae					
*Amitermes amifer*	23727	KX247014	n.d.	n.d.	KX247076
*Amitermes nordestinus*	16373	KX247015	n.d.	n.d.	KX247077
*Cylindrotermes parvignathus*	23881	*DQ442113.1	n.d.	KX247054	KX247075
*Genuotermes spinifer*	16354	KX247016	KY379279	n.d.	KX247078
*Microcerotermes* sp.	21513	KX247013	n.d.	n.d.	KX247074
*Orthognathotermes* sp.	16233	* DQ442213	n.d.	n.d.	KX247073
Syntermitinae					
*Acangaobitermes krishnai*	13670	n.d.	n.d.	n.d.	KX247081
*Armitermes spininotus*	24420	KX247034	n.d.	KX247062	KX247092
*Cahuallitermes intermedius*	15463	KX247037	n.d.	n.d.	n.d.
*Cornitermes acignathus*	24421	KX247039	KX247051	n.d.	n.d.
*Cornitermes bequaerti*	15970	KY379285	KY379284	KX247065	KX247097
*Cornitermes bolivianus*	20596	n.d.	KX247052	KX247067	KY379286
*Cornitermes cumulans*	24423	** EU253899	** EU253860.1	KX247064	KX247096
*Cornitermes ovatus*	20617	KX247038	n.d.	KX247066	KX247098
*Cornitermes silvestrii*	16232	KX247040	KY379283	n.d.	KX247099
*Curvitermes odontognathus*	20705	KX247018	n.d.	n.d.	KX247080
*Cyrilliotermes angulariceps*	20709	KX247027	KY379282	KX247061	n.d.
*Embiratermes brevinasus*	24424	KX247022	n.d.	KX247057	KX247083
*Embiratermes festivellus*	24425	KX247026	KY379281	KX247060	KX247085
*Embiratermes heteropterus*	24427	KX247035	n.d.	n.d.	KX247093
*Embiratermes ignotus*	20810	KX247020	KX247048	KX247055	n.d.
*Embiratermes neotenicus*	23830	KX247025	KX247050	KX247059	n.d.
*Embiratermes silvestrii*	24428	KX247021	KY379280	KX247056	KX247082
*Ibitermes curupira*	24429	KX247029	n.d.	n.d.	KX247087
*Labiotermes emersoni*	16219	KX247030	KY379278	n.d.	KX247088
*Labiotermes labralis*	14771	KX247031	n.d.	n.d.	KX247089
*Labiotermes leptothrix*	20997	KX247032	n.d.	n.d.	KX247090
*Labiotermes orthocephalus*	14829	KX247033	n.d.	n.d.	KX247091
*Macuxitermes triceratops*	16103	n.d.	n.d.	n.d.	KX247095
*Mapinguaritermes peruanus*	14490	KX247028	n.d.	n.d.	KX247086
*Noirotitermes noiroti*	24430	KX247019	n.d.	n.d.	n.d.
*Paracurvitermes manni*	21026	KX247017	KY379277	n.d.	KX247079
*Procornitermes araujoi*	16315	** EU253902	** EU253862	n.d.	n.d.
*Procornitermes lespesii*	24431	KX247041	KX247053	KX247068	n.d.
*Procornitermes triacifer*	24432	n.d.	KY379276	KX247069	KX247100
*Rhynchotermes nasutissimus*	15981	KX247042	n.d.	n.d.	KX247101
*Rhynchotermes perarmatus*	24433	KX247043	KY379275	n.d.	KX247102
*Silvestritermes holmgreni*	20549	KX247023	KX247049	KX247058	n.d.
*Silvestritermes minutus*	20553	KX247024	KY379274	n.d.	KX247084
*Syntermes crassilabrum*	21044	KX247046	KY379273	KX247071	KX247105
*Syntermes grandis*	16338	** EU253903	** EU253863	n.d.	n.d.
*Syntermes molestus*	21069	KX247045	KY379272	KX247070	KX247104
*Syntermes parallelus*	14753	KX247047	n.d.	KX247072	KX247106
*Syntermes spinosus*	21155	KX247044	n.d.	n.d.	KX247103
*Uncitermes teevani*	20574	KX247036	n.d.	KX247063	KX247094

Sequences obtained from other papers are indicated by the asterisks

* [21]

** [23], n.d.: no data.

**Table 2:** Sequences of primers and PCR profiles used.

**Gene**	**Primer**	**Sequence (5' → 3')**			**Reference**	
COI	F-LCO	GGT CAA CAA ATC ATA AAG ATA TTG G			[28]	
	R-HCO	TAA ACT TCA GGG TGA CCA AAA AAT CA			[28]	
COII	F-Leu	TCT AAT ATG GCA GAT TAG TGC			[29]	
	R-Lys	GAG ACC AGT ACT TGC TTT CAG TCA TC			[29]	
Cyt B	cytb612	CCA TCC AAC ATC TCC GCA TGA TGA AA			[30]	
	cytb920	CCC TCA GAA TGA TAT TTG GCC TCA			[30]	
16S rDNA	16SAr	CGC CTG TTT ATC AAA AAC AT			[31]	
	16SF	TTA CGC TGT TAT CCC TAA			[32]	
**Conditions**						
**Gene**	**Heat**	**Denaturation**	**Annealing**	**Extension**	**Final extension**	**Cycles**
COI	94°C (2 min)	94°C (1 min)	43°C (1 min)*	72°C (1 min 15 s)	72°C (7 min)	40
COII	94°C (2 min)	94°C (1 min)	45 to 53°C (1 min)*	72°C (1 min 15 s)	72°C (7 min)	40
Cyt B	94°C (2 min)	94°C (1 min)	50°C (1 min)	72°C (1 min 15 s)	72°C (7 min)	40
16S rDNA	94°C (2 min)	94°C (1 min)	50°C (1 min)	72°C (1 min 15 s)	72°C (7 min)	40

*every 2°C, the temperature was maintained for 30 s.

### Analyses

The saturation of the molecular data was assessed with DAMBE v.6.0.48 [33] using the test of substitution saturation by [34, 35]. The saturation test showed little saturation, indicating that the data were suitable for phylogenetic analysis (Iss < ISSc; p < 0.05).

To evaluate the most useful data set, we made several tests combining different sets of sequences (the four gene sequences, COII + 16S rDNA + COI, COII + 16S rDNA + Cytb and only COII + 16S rDNA), with and without the morphological data, and with the protein-coding sequences partitioned either by genes or by the codon position. The results are summarized in the Table 3.

**Table 3:** Summarized results of combining different sets of sequences and types of codification for protein-coding sequences and their respective Estimated sample sizes (ESS) of each run combined.

	COII; COI; Cytb; 16S rDNA	COII; COI; 16S rDNA	COII; Cytb; 16S rDNA	COII;16S rDNA
Without the morphological data Protein-coding	S13 Fig	S10 Fig	S16 Fig	S6 Fig
sequences partitioned by genes	ESS: 60648	ESS: 63697	ESS: 56201	ESS: 50544
Without the morphological data Protein-coding	S12 Fig	S9 Fig	S15 Fig	S5 Fig
sequences partitioned by the codon position	ESS: 58695	ESS: 60012	ESS:54663	ESS:51510
With morphological data Protein-coding	S11 Fig	S8 Fig	S14 Fig	Fig 18
sequences partitioned by genes	ESS: 56726	ESS: 56635	ESS: 60688	ESS: 65654
With morphological data Protein-coding	Fig 19	S7 Fig	Fig 20	S4 Fig
sequences partitioned by the codon position	ESS: 56656	ESS: 55645	ESS: 56361	ESS: 57063

The models for each DNA data partition were determined using the JModel Test 2 [36] and PartitionFinder v1.1.0 [37], for the morphological data partition the states were unordered. The Bayesian inference analyses were performed with MrBayes version 3.2.1 [38], in the CIPRES Science Gateway V. 3.3 [39]; in all analyses, four chains were run for 50 million generations and sampled every thousand generations (two runs). In all cases the burn-in limitation was determined by visual inspection of the trace-plot and evaluation of the effective sample size value (ESS) of the combined runs, using Tracer v1.6 [40]. The burn-in of 1% was sufficient.

Ancestral character states were reconstructed with the help of Mesquite v.3.04 [24], by the parsimony criterion.

## Results and discussion

From the total of 48 species used in our analyses, we obtained DNA data from three or four different gene for 25 species, two different sequences for 16 species and only one sequence for 4 species (Table 1). Two taxa are represented only by morphological data. About one third of the sequences information is absent. Although the poverty of sequences may compromise the results, the majority of taxa share COII and 16s rDNA information (The information for COII sequences is absent only in four taxa and for 16S rDNA in 10), the major part of lacking information is concentrated in COI and Cytb sequences.

**Fig 18:**
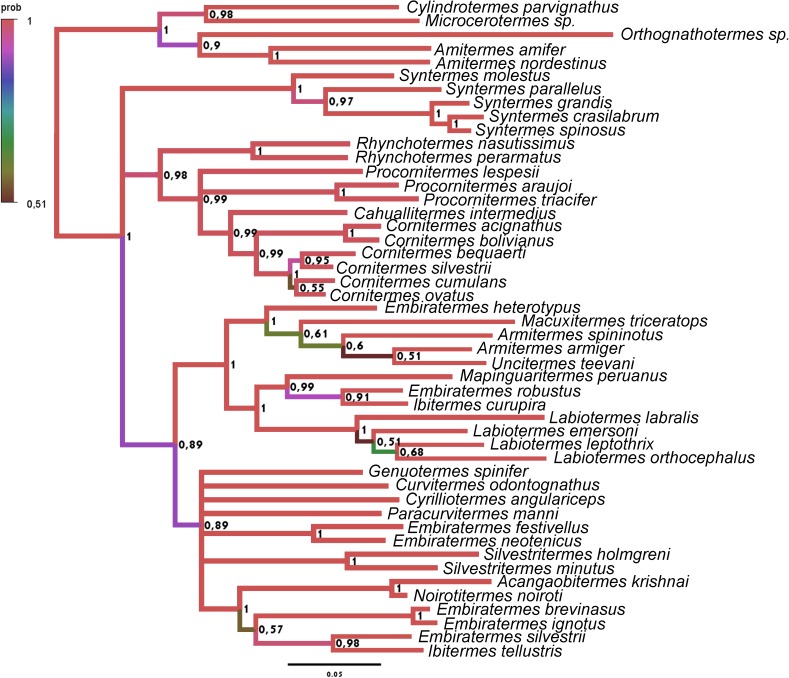
Tree obtained with the Bayesian analysis with morphological data and COII, 16S rDNA sequences, partitioned by genes. The respective posterior probability is indicated above each node, and the branch color represents the posterior probability.

**Fig 19:**
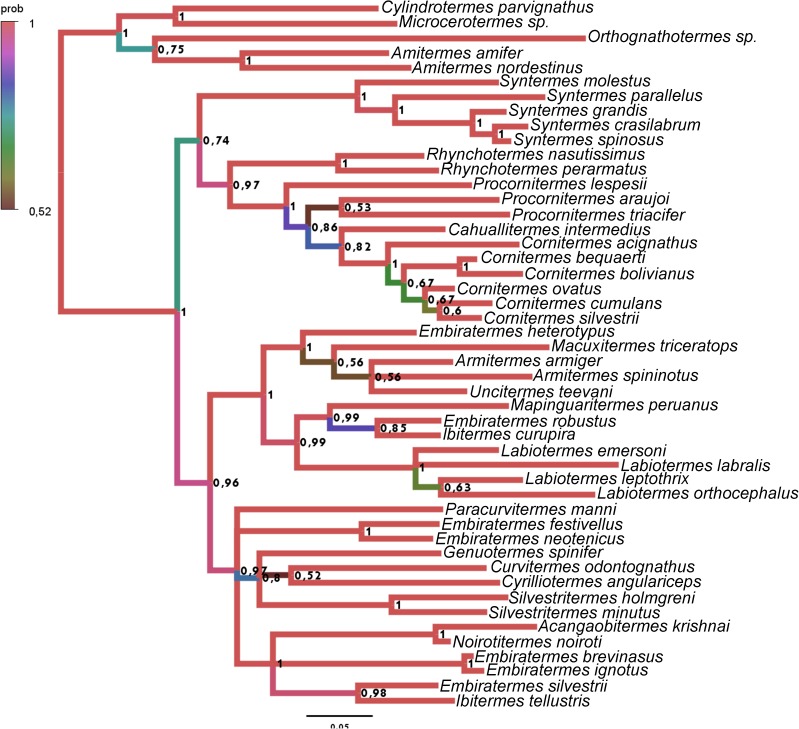
Tree obtained with the Bayesian analysis with morphological data and all four sequences, partitioned by codons. The respective posterior probability is indicated above each node, and the branch color represents the posterior probability.

**Fig 20:**
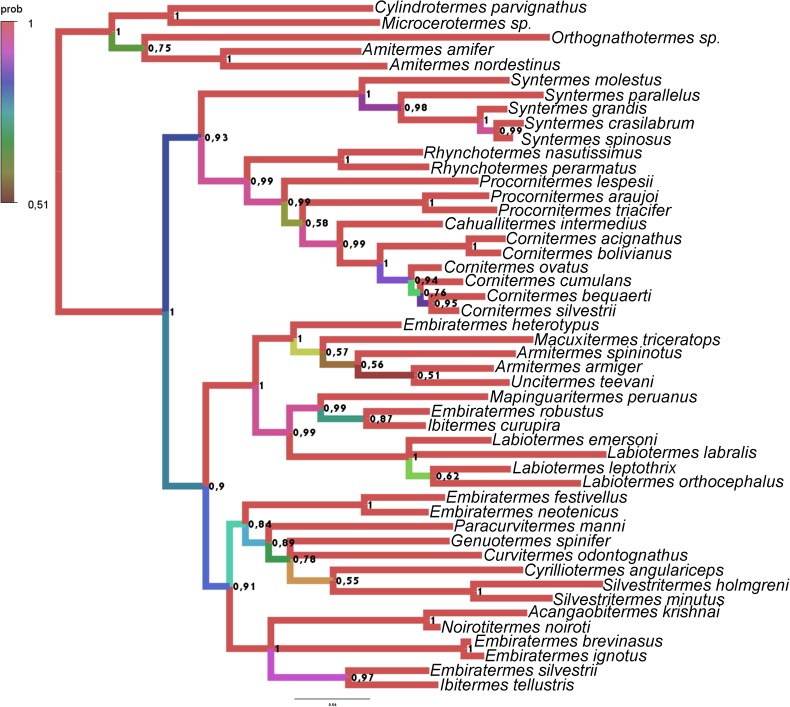
Tree obtained with the Bayesian analysis with morphological data and COII, Cytb, 16S rDNA sequences, partitioned by codons. The respective posterior probability is indicated above each node, and the branch color represents the posterior probability.

Among the trees obtained, three of them present informative topology (with few polytomies) and high posterior probabilities (especially in the basal nodes); 1)The result of an analysis with Morphology + COII + 16S rDNA, partitioned by genes (COII: GTR +I + G, 16S rDNA: GTR +G) represented in Fig 18 and Fig 20A (29 nodes more than 0.9, 2 nodes equal 0.89, and 7 nodes less than 0.8); 2) The result of Morphology + all sequences, with protein-coding genes partitioned by codons (1st codon: GTR+G, 2nd codon: HKY+I+G, 3rd codon: GTR+I+G, 16S rDNA: GTR+I+G), represented in Fig 19 and Fig 20B (26 nodes more than 0.9, 4 nodes between 0.8 and 0.9, and 10 nodes less than 0.8); 3) The result of Morphology COII + 16S rDNA + Cytb, with protein-coding genes partitioned by codons (1st codon: SYM+I+G, 2nd codon: HKY+I+G, 3rd codon: GTR+I+G, 16S rDNA: GTR+I+G), represented in Fig 20 and Fig 20C (30 nodes more than 0.9, 3 nodes between 0.8 and 0.9, and 9 nodes less than 0.8). Their respective traceplots are illustrated in S1–S3 Figs. Considering all obtained trees (Figs 18–20 and S4–S16 Figs), the exclusion of morphological data from the analysis result in large pectinate nodes (S5, S6, S9, S10, S12, S13, S15 and S16 Figs).

**Fig 21:**
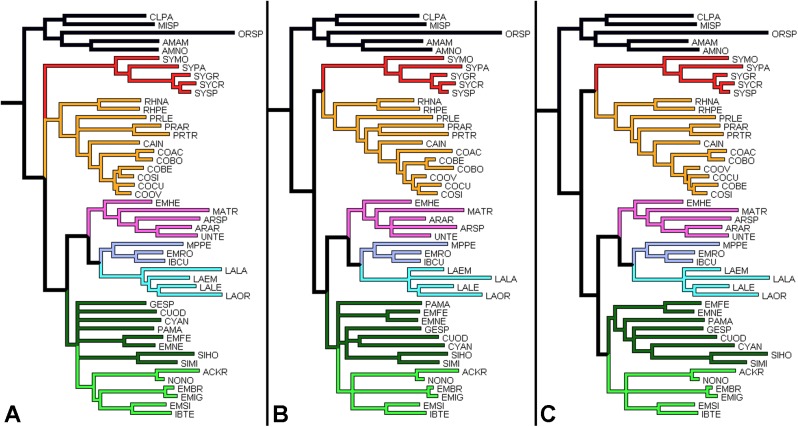
Comparison between topologies of the more consistent trees. A. analysis with morphological data and COII, 16S rDNA sequences, partitioned by genes; B. analysis with morphological data and all sequences, partitioned by codons; C. analysis with morphological data and COII, Cytb, 16S rDNA sequences, partitioned by codons. The equivalent branches are indicated by the colors; the name of each species is represented by an acronym.

The position of *Acangaobitermes krishnai*, *Cahuallitermes intermedius*, *Macuxitermes triceratops* and *Noirotitermes noiroti* represented by only one sequence and *Armitermes armiger* and *Ibitermes tellustris*, with no sequences, remains stable in the three more consistent trees (Fig 21), and considering just morphological characters, mainly from internal morphology, the position among them seems reliable.

Comparing the three most robust results (Fig 21A–21C), their topologies have a few divergences; the groups of genera (delimited by the best-supported basal nodes, and indicated by the same colors in each tree) were recovered with identical taxa compositions.

The more notable differences among the selected trees are: The position of the genus *Syntermes* (the red branch, with taxa initiated by the acronym “SY”), not resolved in tree A and positioned in trees B and C as a sister group of the yellow branch, composed of *Rhynchotermes* (indicated by the acronym “RH”), *Procornitermes* (PR), *Cahuallitermes* (CA) and *Cornitermes* (CO). The relationships among the taxa are indicated by dark-green branches; *Genuotermes* (GESP), *Curvitermes* (CUOD), *Embiratermes* (EM), *Paracurvitermes* (PAMA), *Cyrilliotermes* (CYAN) and *Silvestritermes* (SI); possible paraphyletic in trees A and B, and recovered as a monophyletic group in tree C.

Although these results do not contradict each other, for prudence we opted to reconstruct and discuss the ancestral character states using tree A, which is less resolved but more conservative.

### Taxonomic discussion

The sample of species is sufficiently comprehensive to allow a discussion that previous studies did not attempt. Four genera appeared as paraphyletic in our analysis: *Armitermes* (possibly), *Procornitermes* (possibly), *Embiratermes*, and *Ibitermes*.

The cases of *Armitermes* (pink branch, Fig 21) and *Procornitermes* (part of the yellow branch, Fig 21) can be resolved with reallocation of a few species: for *Armitermes* the most conservative solution is include all taxons (*Armitermes*, *Macuxitermes*, *Uncitermes* and *Embiratermes heterotypus*) into a single genus, since the node that it is grouping the five taxa has a high posterior probability. Nevertheless, considering we only obtain one sequences for *Macuxitermes*, and also the support for the three more internal nodes are low, we think more studies are necessary to infer consistently the relationship among the taxa before introducing nomenclatural changes; for *Procornitermes*, resurrecting it from *Triacitermes* Emerson, now including *Procornitermes triacifer* and *P*. *araujoi* is a admissible solution, however the paraphyly of the genus is not a consensus among the results. More detailed studies for these cases are necessary before formal proposals for nomenclatural changes can be made.

A revisionary work is necessary in the next future to reassess generic and specific limits as well as the intergeneric relationships of *Embiratermes* and *Ibitermes* within other members of the subfamily, since their named species used herein as terminals are spread all over the tree.

A surprising result is the position of *Genuotermes*, deeply inserted in our tree. Although the association with Syntermitinae is unintuitive, some unique characteristics are shared with Syntermitinae. The soldier frontal-gland aperture is at the tip of a large projection located in the frontal region of the head; the soldier mandibles have a clearly recognizable molar plate and prominence, as in *Silvestritermes* [3], *Cyrilliotermes* [5], and *Curvitermes* [6]; and the worker gut morphology is very similar, including the characteristic dilated P1 of Syntermitinae [41]. Considering these points, the reallocation of the genus to Syntermitinae is expected, following comprehensive studies of other Neotropical termitine genera.

### Defense and feeding behavior in Syntermitinae

Two aspects stand out in termite research: defense and feeding habits. The first aspect relates to the soldier caste in Isoptera, which comprises a very particular case for evolutionary biology. Soldiers are a “burden” on the colony maintenance, since they need to be fed by the workers, and the effective contribution of a very specialized caste for the colony defense is not clear. The proportion of soldiers and workers varies widely among species [42] and nearly 10% of termite species do not have soldiers (mainly Apicotermitinae). The second aspect relates to the central role of termites as decomposers in tropical climates; they can comprise as much as 95% of the soil insect biomass [43]. Termites can obtain nourishment from a variety of plant biomass sources, including wood, rotting wood, grass, cultures of fungi, lichen and humus; and this diversification of feeding habits appears to be linked to termite species diversification [21].

**Fig 22:**
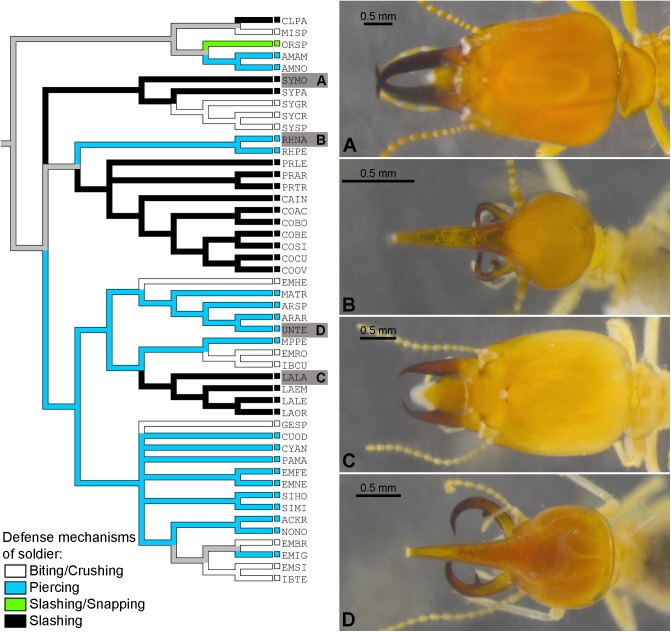
Reconstruction of the primary individual defense mechanisms of syntermitine soldiers. Examples of soldier head shapes, A. *Syntermes molestus*; B. *Rhynchotermes nasutissimus*; C. *Labiotermes labralis*; D. *Uncitermes teevani*; the state of each taxon is indicated by the color of the squares, and the name of each species is represented by an acronym.

For the reconstruction of the defense behavior, each taxon was classified according to the categories of primary individual defense mechanisms summarized in [44]. Three categories of defense were recognized: “Biting/Crushing” (example in Fig 3A) “Piercing” (examples in Fig 3B and 3F), and “Slashing” (examples in Fig 3C, 3D and 3E). *Orthognathotermes* sp. is formally classified as “Slashing/Snapping”, but this is not relevant to the present discussion; the result is represented in Fig 22. The reconstruction showed that equivalent categories of defense evolved independently several times in syntermitine history: “Slashing” mandibles appeared two or three times independently (Fig 22, black branches), “Piercing” (Fig 22, blue branches) two or three times, and “Biting/Crushing” (Fig 22, white branches) five or six times.

Fig 22 shows two cases that well represent the degree of convergence of form. The soldier head of *Rhynchotermes nasutissimus* (Fig 22B) is clearly similar to *Uncitermes teevani* (Fig 22D), although *U*. *teevani* is more kindred to *Labiotermes labralis* (Fig 22C), which itself shares several traits with a distantly related species, *Syntermes molestus* (Fig 22A). These are not the only cases of convergence; the species of *Embiratermes* share a variety of external traits and are spread among four clades. It is unnecessary to exhaustively discuss all the cases, which would excessively lengthen this article. The soldier external morphology of each genus can be found in revisionary studies, or in the identification keys of Constantino [45, 46].

Other cases of convergence in termite soldier morphology were discussed by Inward and collaborators [21], who found that the asymmetrical snapping mandibles, a very specialized type of termite defense, evolved independently four times among all Isoptera. In the present case, we found a high degree of convergence in the soldier types of defense inside a much more restricted group.

The means of establishing the diet of each termite species can be controversial. Some specialists have proposed using analyses of the gut contents [47] or nitrogen stable-isotope ratios [48], but no discrete criteria have been developed to classify the termite diet precisely. Despite this, the resources consumed by termites can be organized in a continuous humification gradient, from wood and grass, which are non-humified resources, at one extreme; and very humified resources, such as humus and stercoral material from other nests, at the other [49]. This gradient can be correlated and recognized in the worker mandible morphology [50, 51]. Species that feed on non-humified resources have the molar region with conspicuous ridges and a relatively small apical tooth, which is termed “xylophagous morphology” (Fig 17A and 17B, for example). Species that feed on humified resources have the molar region without ridges and a prominent apical tooth, termed “intermediate/geophagous morphology” (Fig 17C–17E, for example).

**Fig 23:**
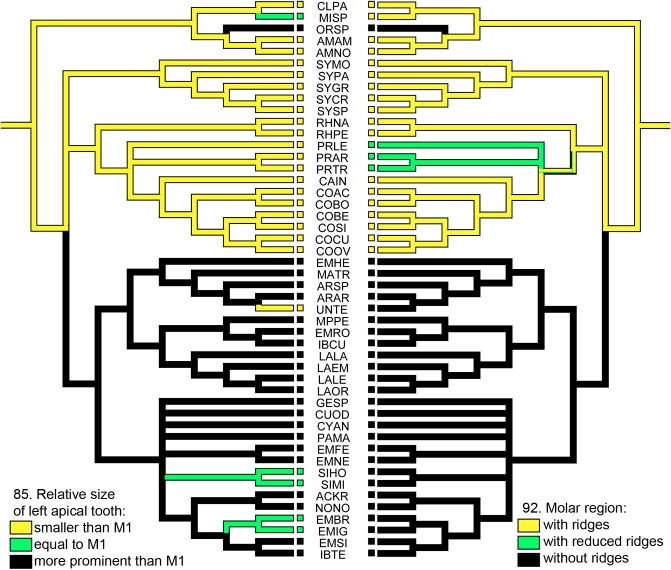
Reconstruction of the syntermitine mandible characters. Molar region (character 92) and relative size of left apical tooth (character 85) reconstructions. The state of each taxon is indicated by the color of the squares, and the name of each species is represented by an acronym.

The reconstruction of these two characters (Fig 23), relative size of the left apical tooth (85) and the molar region (92), showed the expected overlap between these characteristics; xylophagous traits are traced in yellow and geophagous in black.

Our topology indicated that in the syntermitine evolutionary history, a very early split occurred between lineages that tend to feed on non-humified resources and species that tend to feed on very humified resources. The change in the species’ diet was reflected in more than the mandible shape, and a complex change in the digestive apparatus and the associated symbionts would be expected; however, knowledge of Termitidae digestive processes and their correlation with the gut morphology is presently limited. We expect that the Syntermitinae will provide a useful and more practical case for future studies.

Unfortunately, the lack of syntermitine fossils limits the dating and evolutionary interpretations of these characteristics. The oldest record is an ichnofossil, described as a *Syntermes*-like nest [52], from southern Argentina and dating from the late Early Miocene; all other syntermitine fossil records in the literature are much more recent [53, 54].

## Supporting information

S1 Table Character Matrix. (XLSX)

S1 Fig Traceplots of the analysis with morphological data and COII, 16S rDNA sequences, partitioned by genes. The two firsts plots correspond to each run and the low to the combined result.
(TIF)

S2 Fig Traceplots of the analysis with morphological data and all four sequences, partitioned by codons. The two first plots correspond to each run and the low to the combined result.
(TIF)

S3 Fig Traceplots of the analysis with morphological data and COII, Cytb, 16S rDNA sequences, partitioned by codons. The two first plots correspond to each run and the low to the combined result.
(TIF)

S4 Fig Tree obtained with the Bayesian analysis with morphological data and COII, 16S rDNA sequences, partitioned by codons. The respective posterior probability is indicated above each node, the branch color represents the posterior probability.
(TIF)

S5 Fig Tree obtained with the Bayesian analysis with COII and 16S rDNA sequences, partitioned by codons. The respective posterior probability is indicated above each node, the branch color represents the posterior probability.
(TIF)

S6 Fig Tree obtained with the Bayesian analysis with COII and 16S rDNA sequences, partitioned by genes. The respective posterior probability is indicated above each node, the branch color represents the posterior probability.
(TIF)

S7 Fig Tree obtained with the Bayesian analysis with morphological data and COII, COI, 16S rDNA sequences, partitioned by codons. The respective posterior probability is indicated above each node, the branch color represents the posterior probability.
(TIF)

S8 Fig Tree obtained with the Bayesian analysis with morphological data and COII, COI, 16S rDNA sequences, partitioned by genes. The respective posterior probability is indicated above each node, the branch color represents the posterior probability.
(TIF)

S9 Fig Tree obtained with the Bayesian analysis with COII, COI and 16S rDNA sequences, partitioned by codons. The respective posterior probability is indicated above each node, the branch color represents the posterior probability.
(TIF)

S10 Fig Tree obtained with the Bayesian analysis with COII, COI and 16S rDNA sequences, partitioned by genes. The respective posterior probability is indicated above each node, the branch color represents the posterior probability.
(TIF)

S11 Fig Tree obtained with the Bayesian analysis with morphological data and all four sequences, partitioned by genes. The respective posterior probability is indicated above each node, the branch color represents the posterior probability.
(TIF)

S12 Fig Tree obtained with the Bayesian analysis with all four sequences, partitioned by codons. The respective posterior probability is indicated above each node, the branch color represents the posterior probability.
(TIF)

S13 Fig Tree obtained with the Bayesian analysis with all four sequences, partitioned by genes. The respective posterior probability is indicated above each node, the branch color represents the posterior probability.
(TIF)

S14 Fig Tree obtained with the Bayesian analysis with morphological data and COII, Cyt b, 16S rDNA sequences, partitioned by genes. The respective posterior probability is indicated above each node, the branch color represents the posterior probability.
(TIF)

S15 Fig Tree obtained with the Bayesian analysis with COII, Cyt b, 16S rDNA sequences, partitioned by codons. The respective posterior probability is indicated above each node, the branch color represents the posterior probability.
(TIF)

S16 Fig Tree obtained with the Bayesian analysis with COII, Cyt b, 16S rDNA sequences, partitioned by genes. The respective posterior probability is indicated above each node, the branch color represents the posterior probability.
(TIF)

S1 File Additional information about the material used for DNA extractions in this work. (DOCX)
