# Supplementary figures and images for: Phylogenetic reconstruction of Syntermitinae (Isoptera, Termitidae) based on morphological and molecular data
Source: PLoS One. 2017 Mar 22;12(3):e0174366. doi: 10.1371/journal.pone.0174366 (PMC5362239; doi:10.1371/journal.pone.0174366)

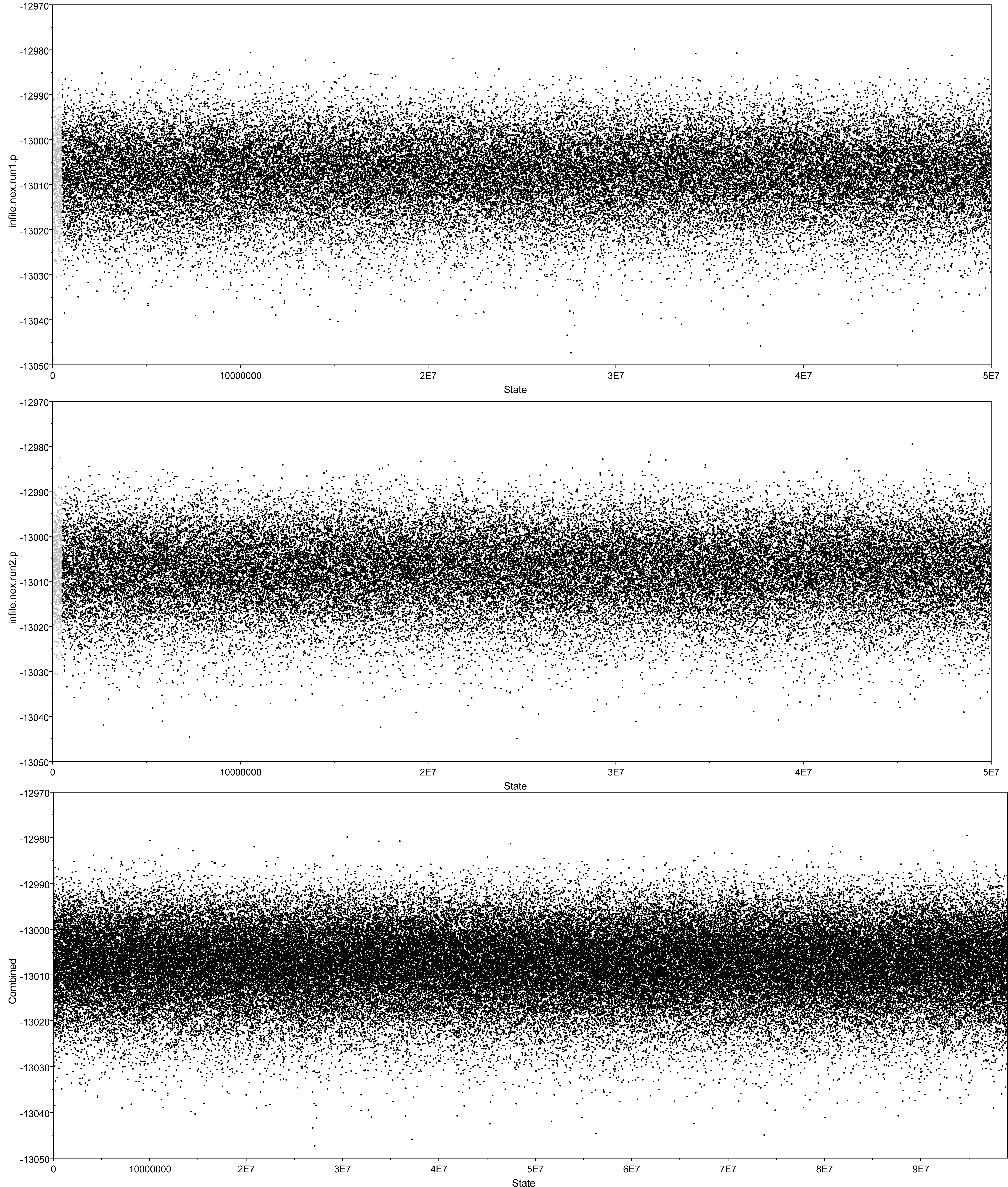

Supplement: S1 Fig — The two firsts plots correspond to each run and the low to the combined result. (TIF) [file pone.0174366.s002.tif]

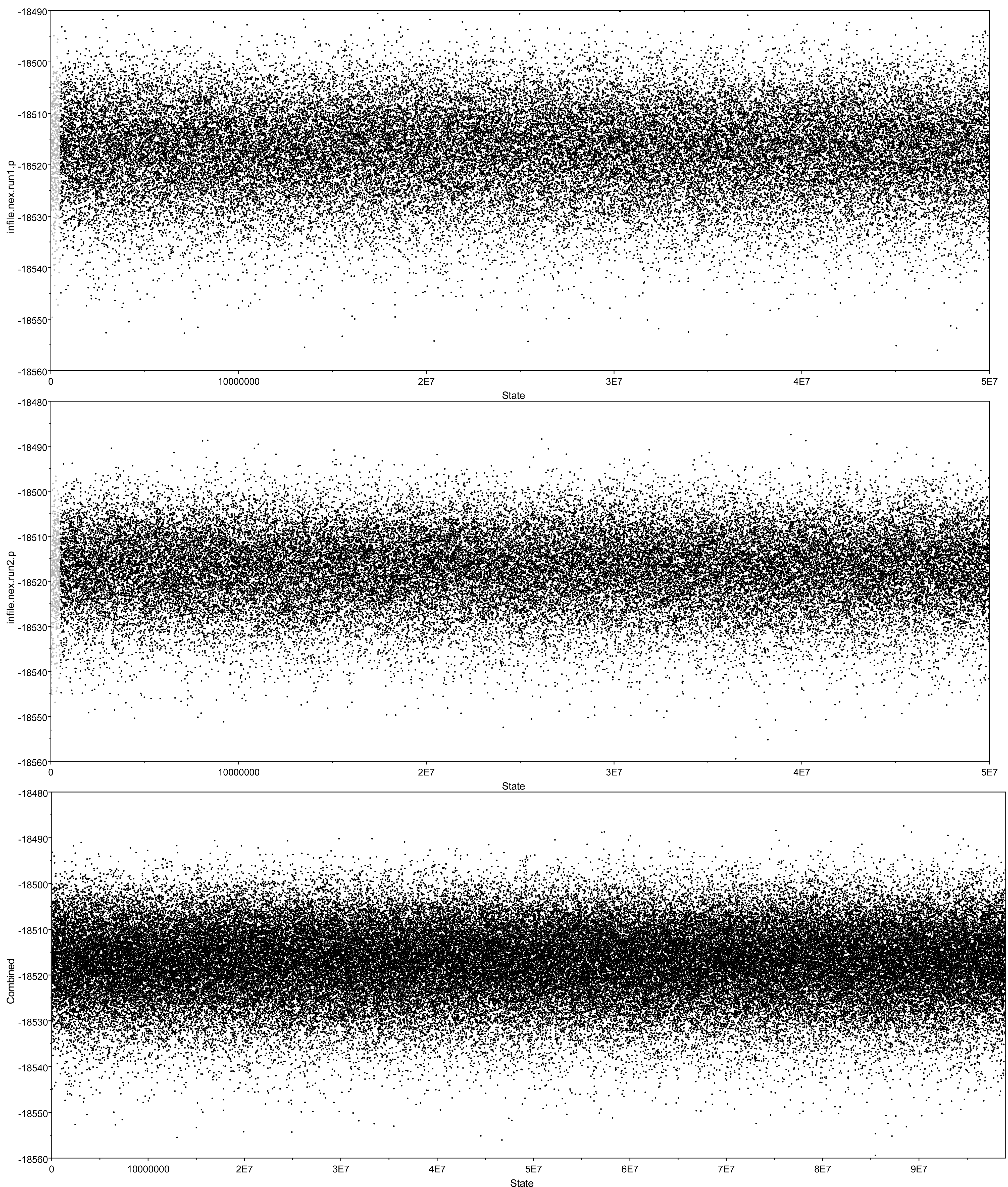

Supplement: S2 Fig — The two first plots correspond to each run and the low to the combined result. (TIF) [file pone.0174366.s003.tif]

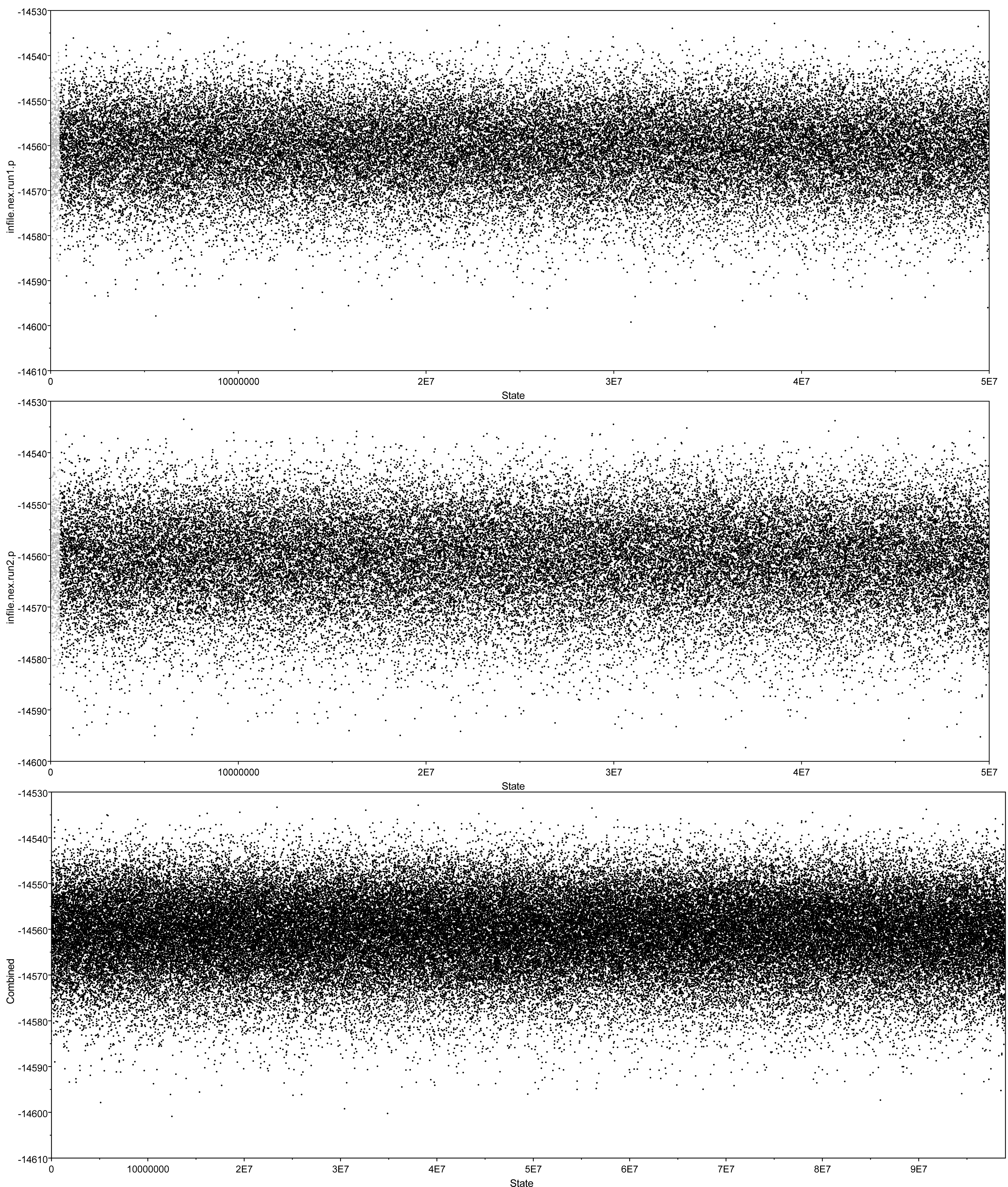

Supplement: S3 Fig — The two first plots correspond to each run and the low to the combined result. (TIF) [file pone.0174366.s004.tif]

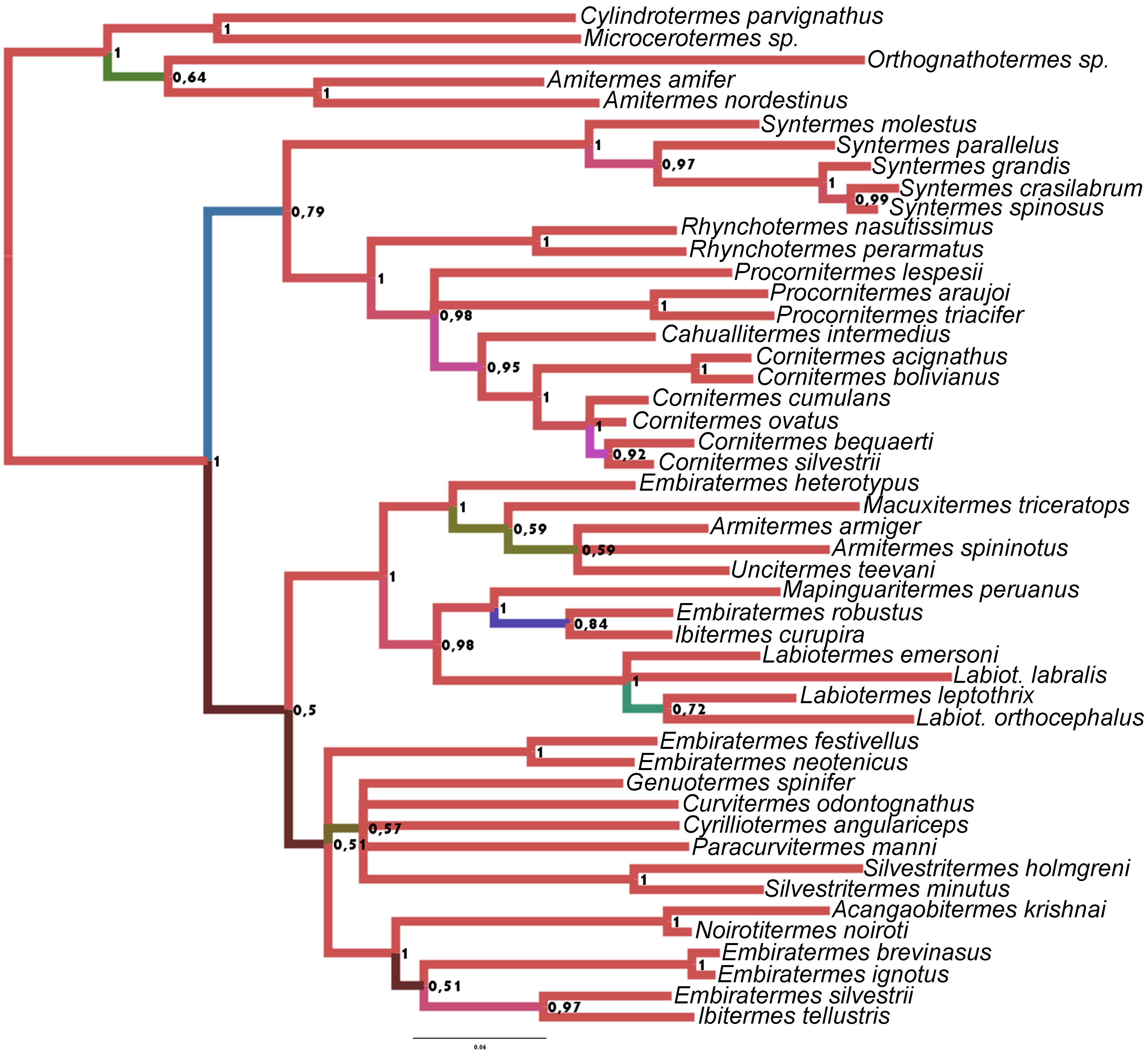

Supplement: S4 Fig — The respective posterior probability is indicated above each node, the branch color represents the posterior probability. (TIF) [file pone.0174366.s005.tif]

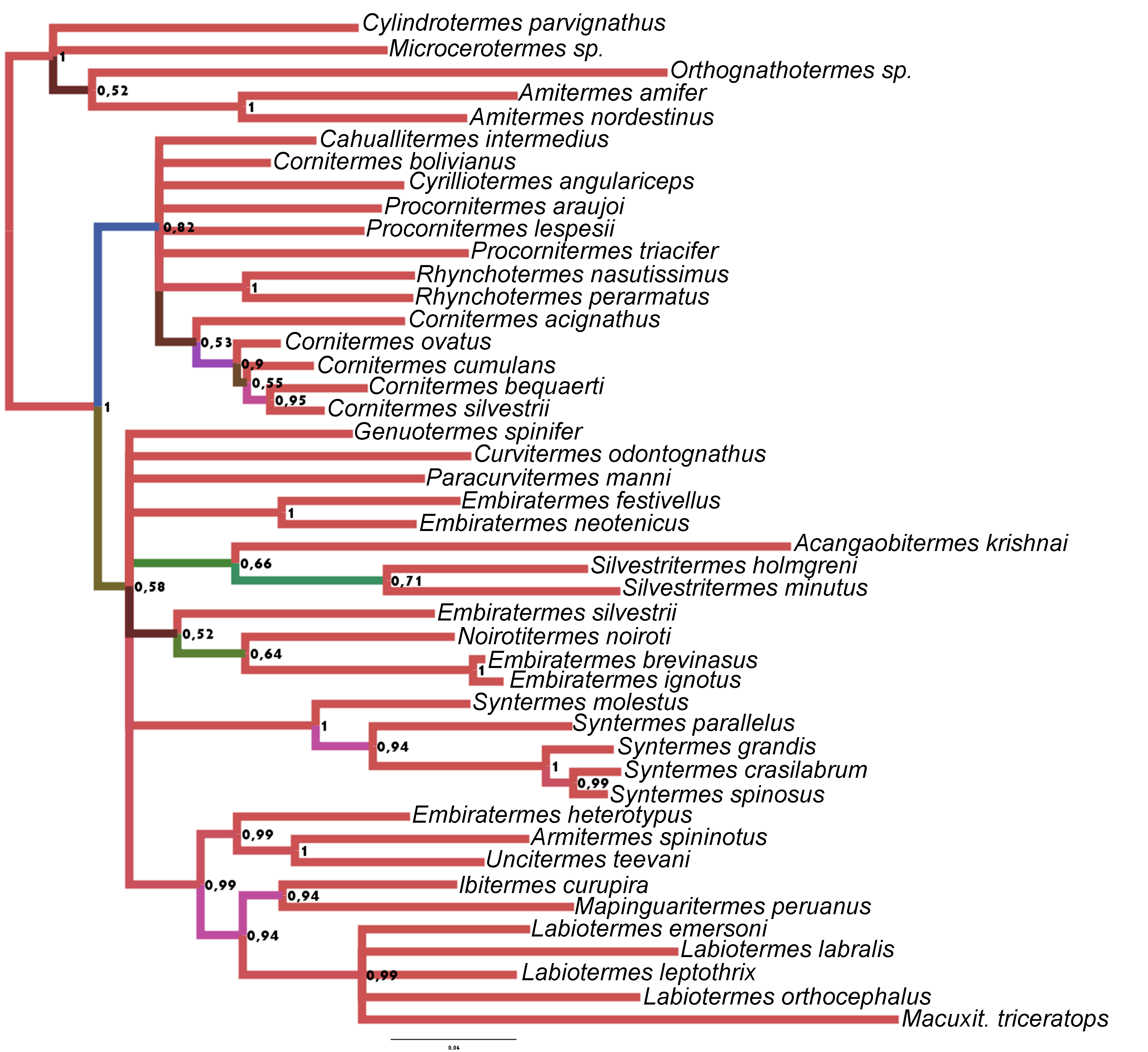

Supplement: S5 Fig — The respective posterior probability is indicated above each node, the branch color represents the posterior probability. (TIF) [file pone.0174366.s006.tif]

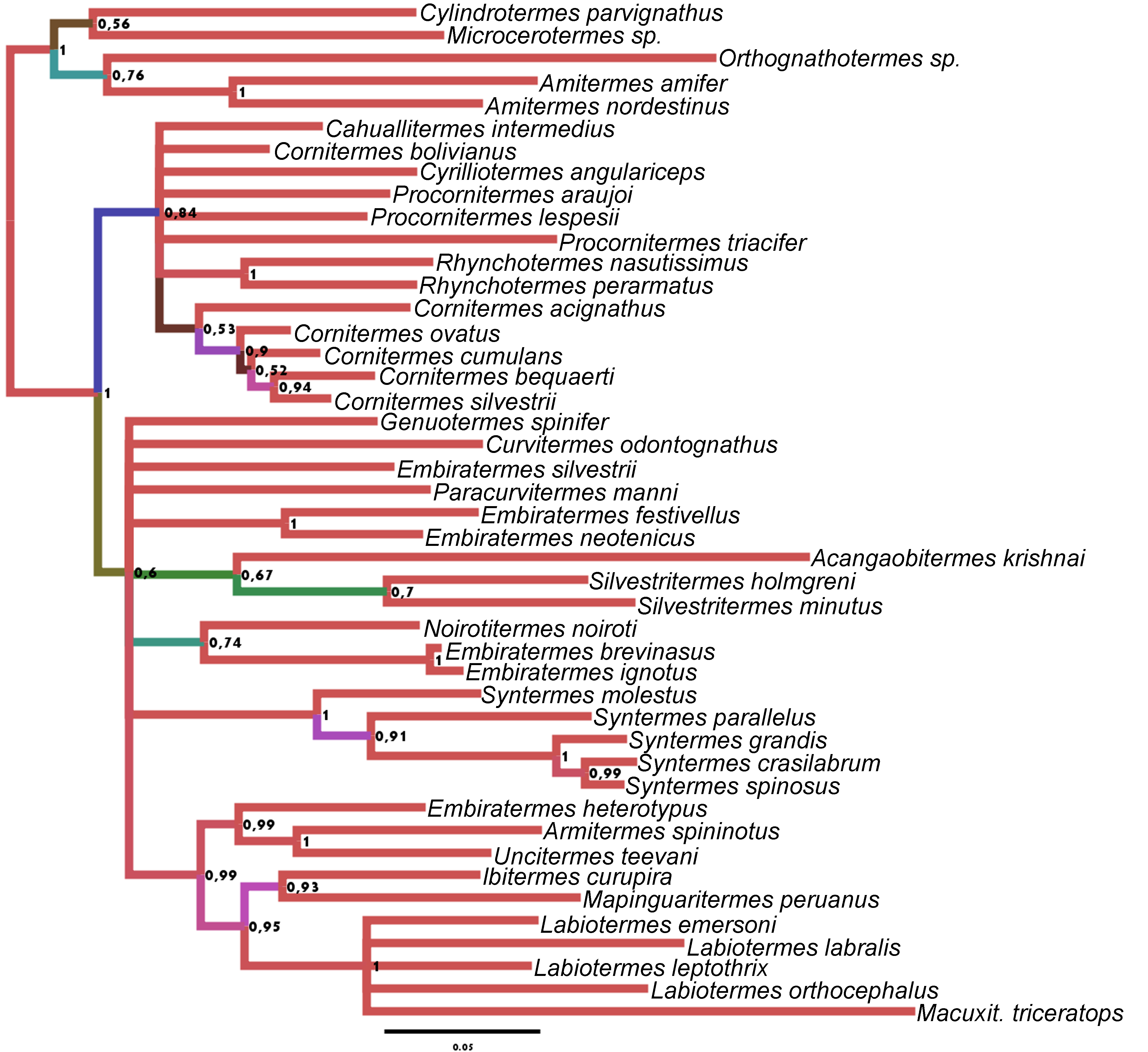

Supplement: S6 Fig — The respective posterior probability is indicated above each node, the branch color represents the posterior probability. (TIF) [file pone.0174366.s007.tif]

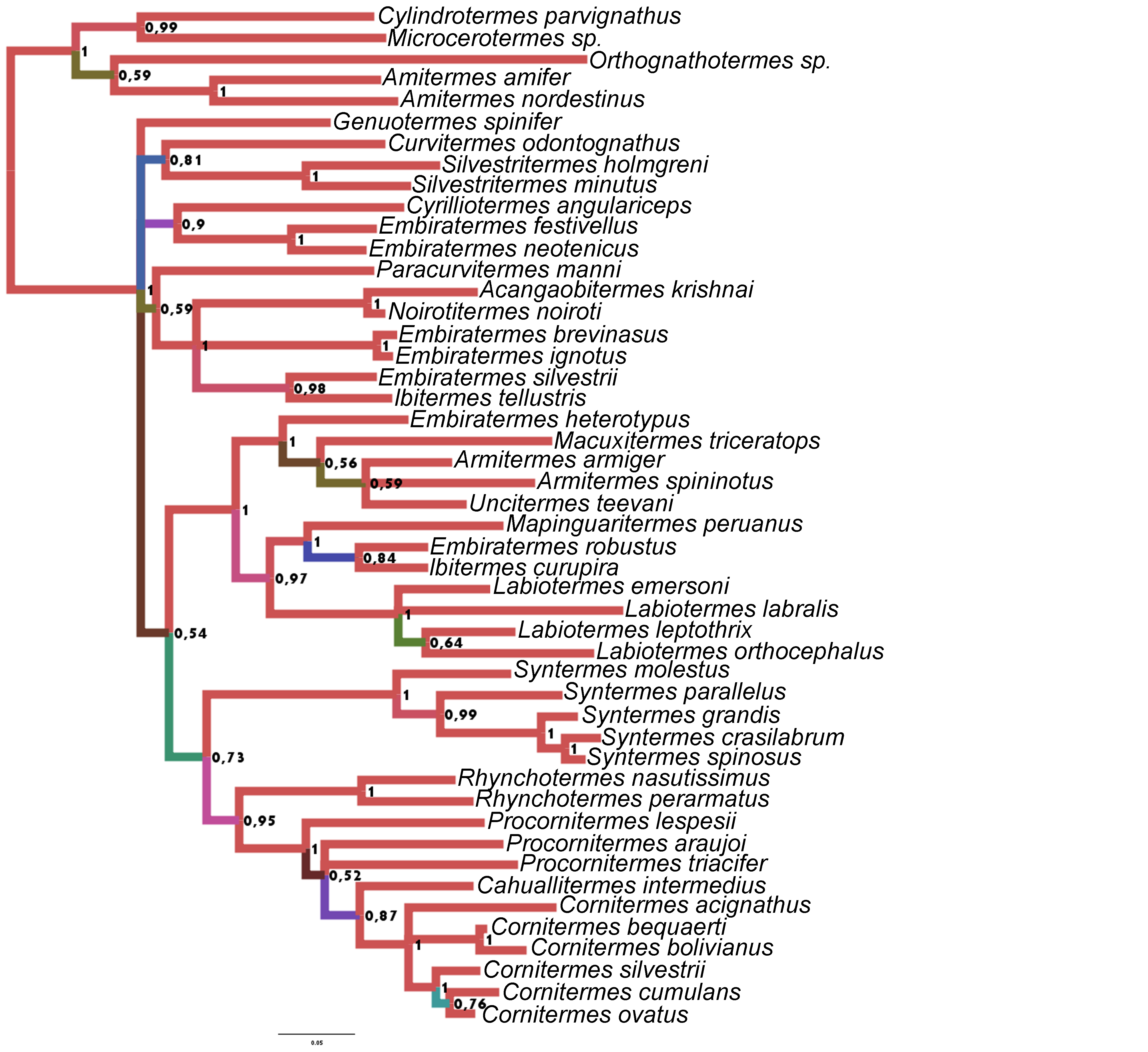

Supplement: S7 Fig — The respective posterior probability is indicated above each node, the branch color represents the posterior probability. (TIF) [file pone.0174366.s008.tif]

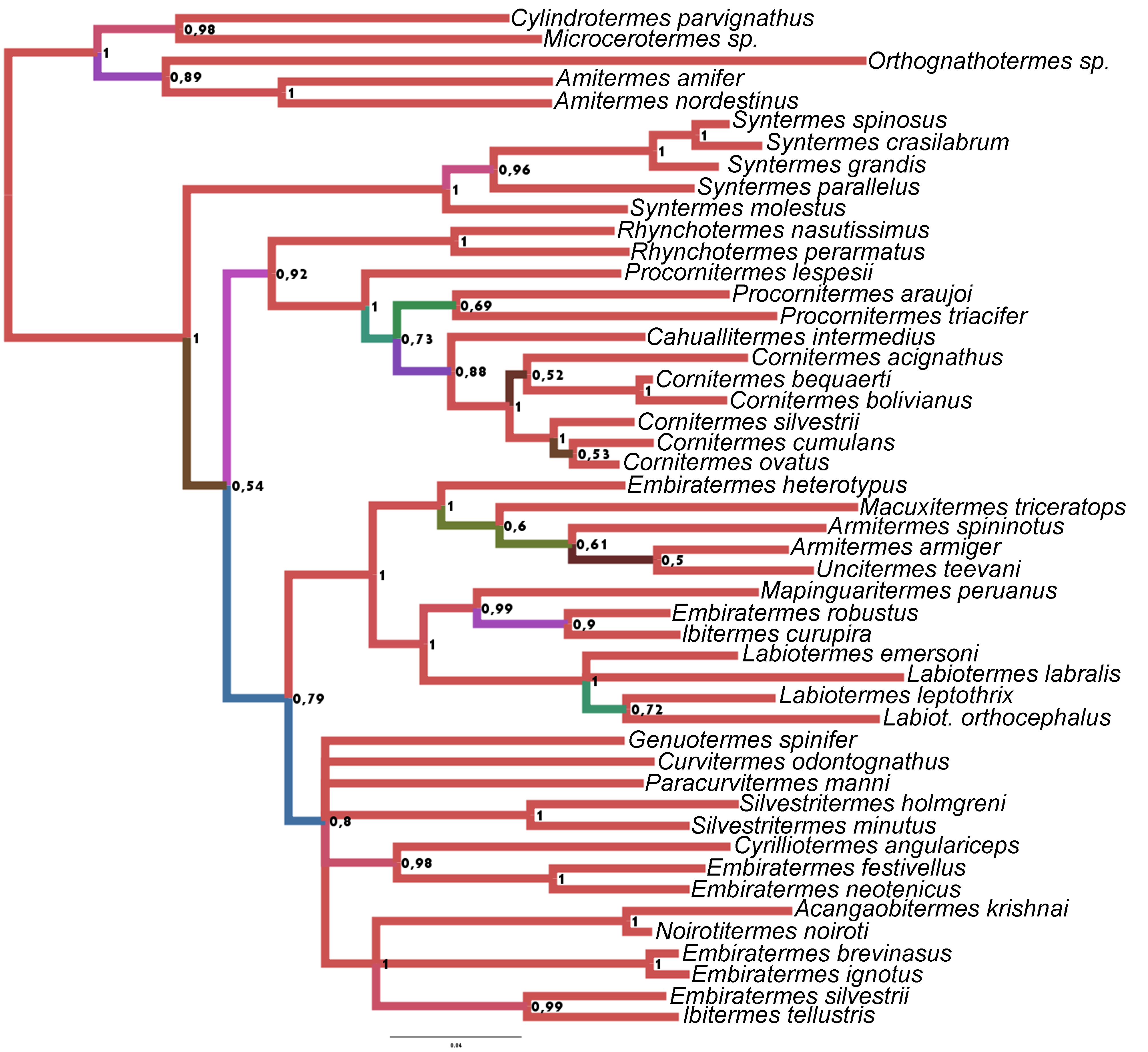

Supplement: S8 Fig — The respective posterior probability is indicated above each node, the branch color represents the posterior probability. (TIF) [file pone.0174366.s009.tif]

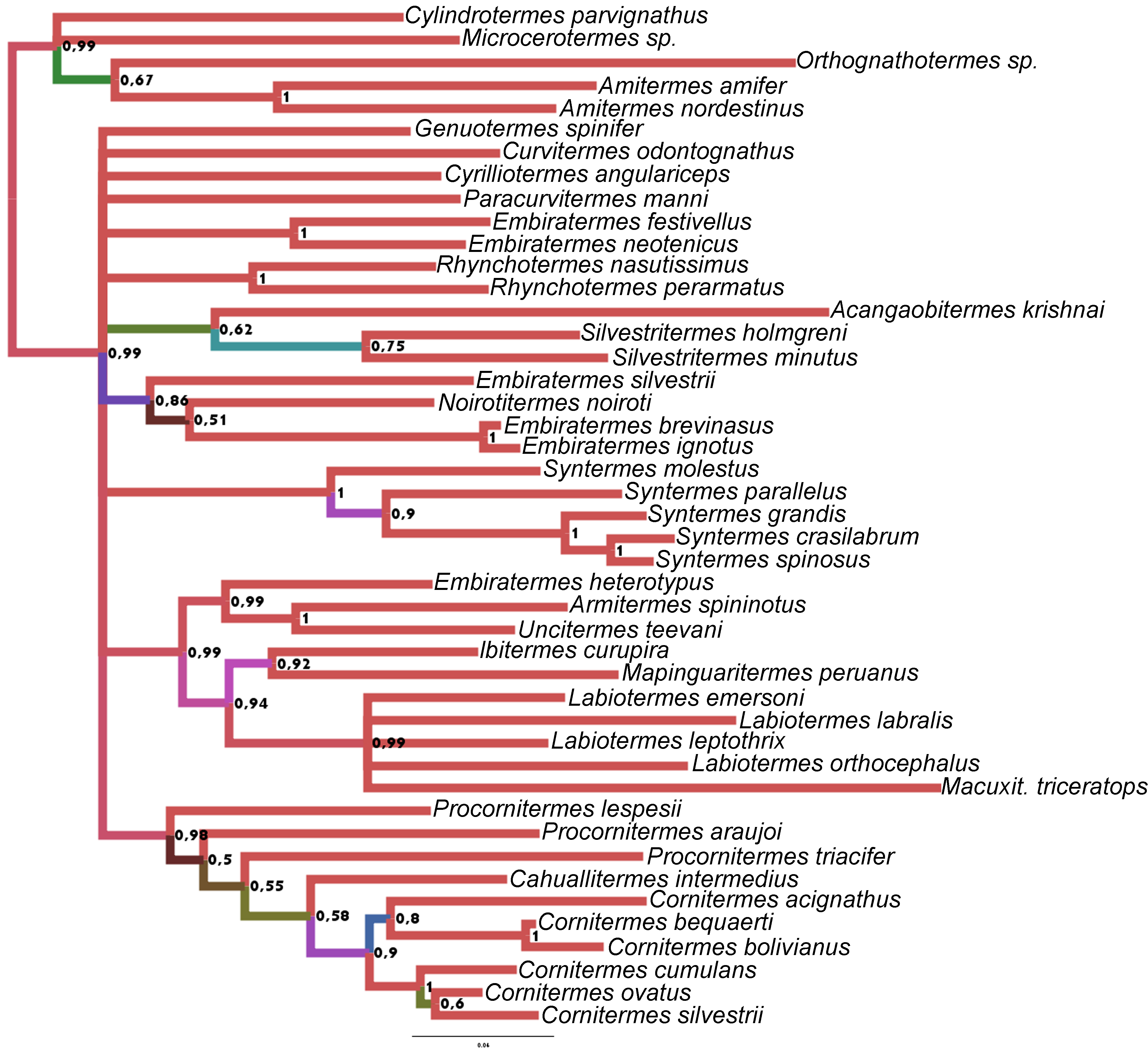

Supplement: S9 Fig — The respective posterior probability is indicated above each node, the branch color represents the posterior probability. (TIF) [file pone.0174366.s010.tif]

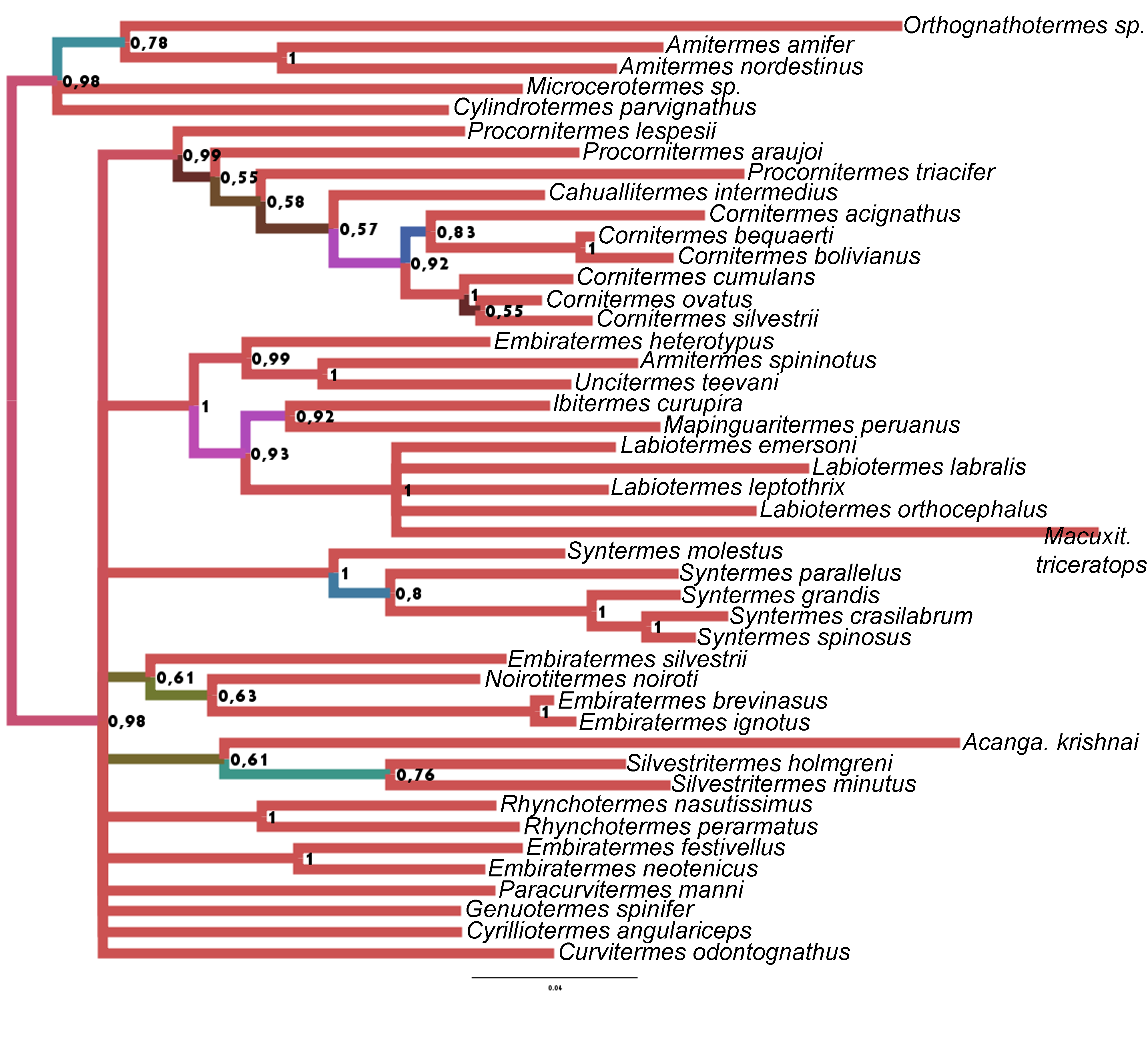

Supplement: S10 Fig — The respective posterior probability is indicated above each node, the branch color represents the posterior probability. (TIF) [file pone.0174366.s011.tif]

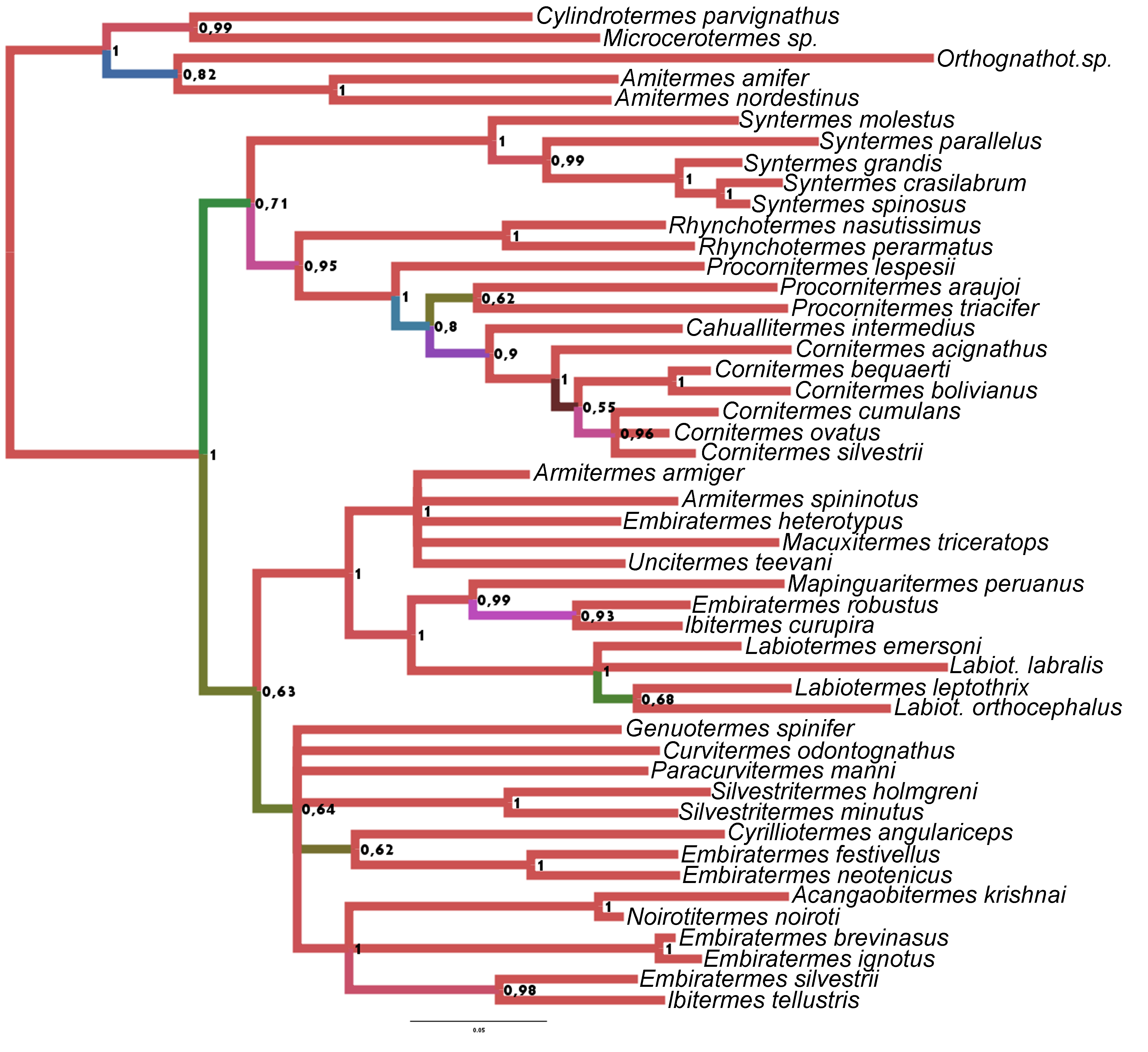

Supplement: S11 Fig — The respective posterior probability is indicated above each node, the branch color represents the posterior probability. (TIF) [file pone.0174366.s012.tif]

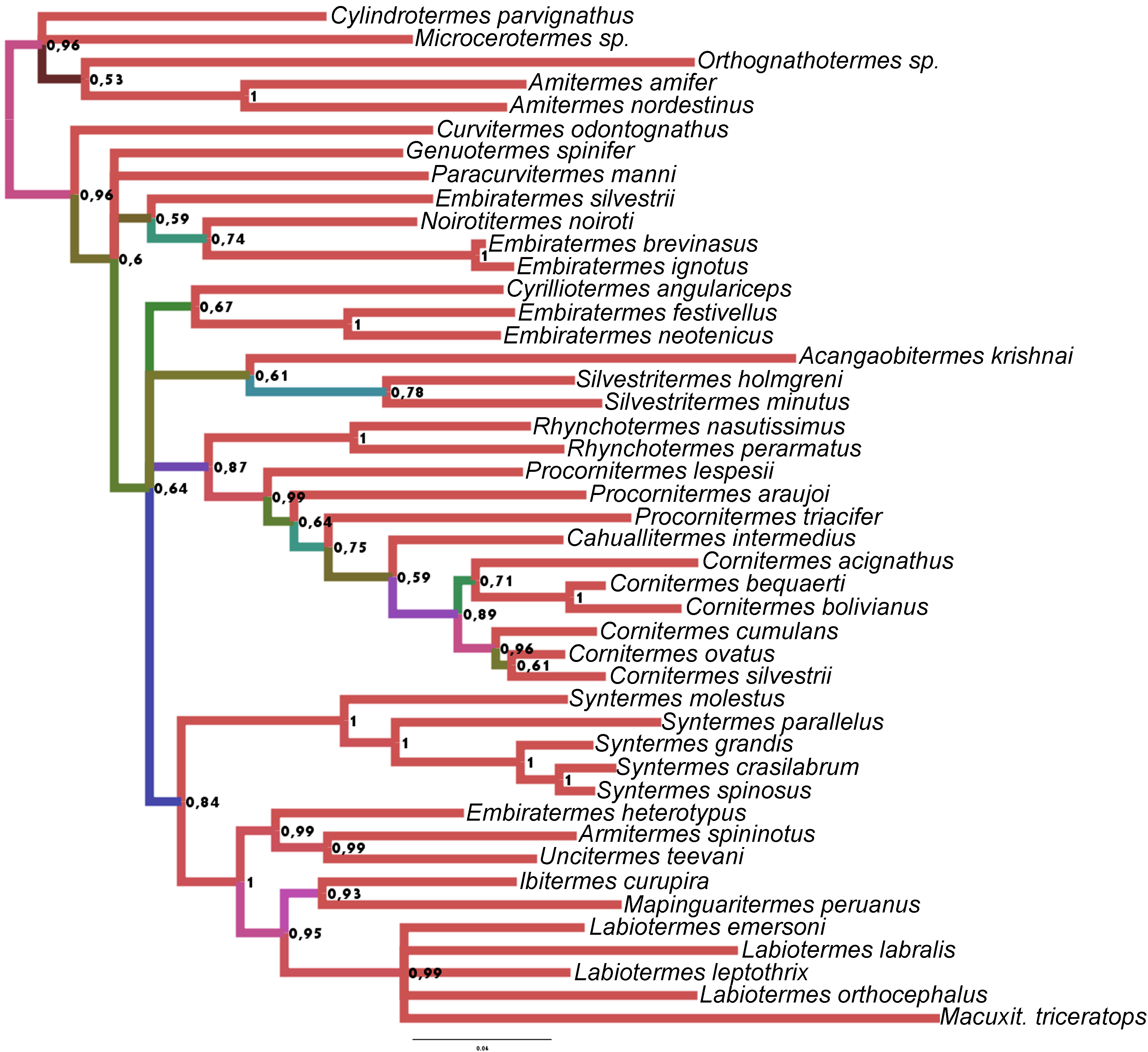

Supplement: S12 Fig — The respective posterior probability is indicated above each node, the branch color represents the posterior probability. (TIF) [file pone.0174366.s013.tif]

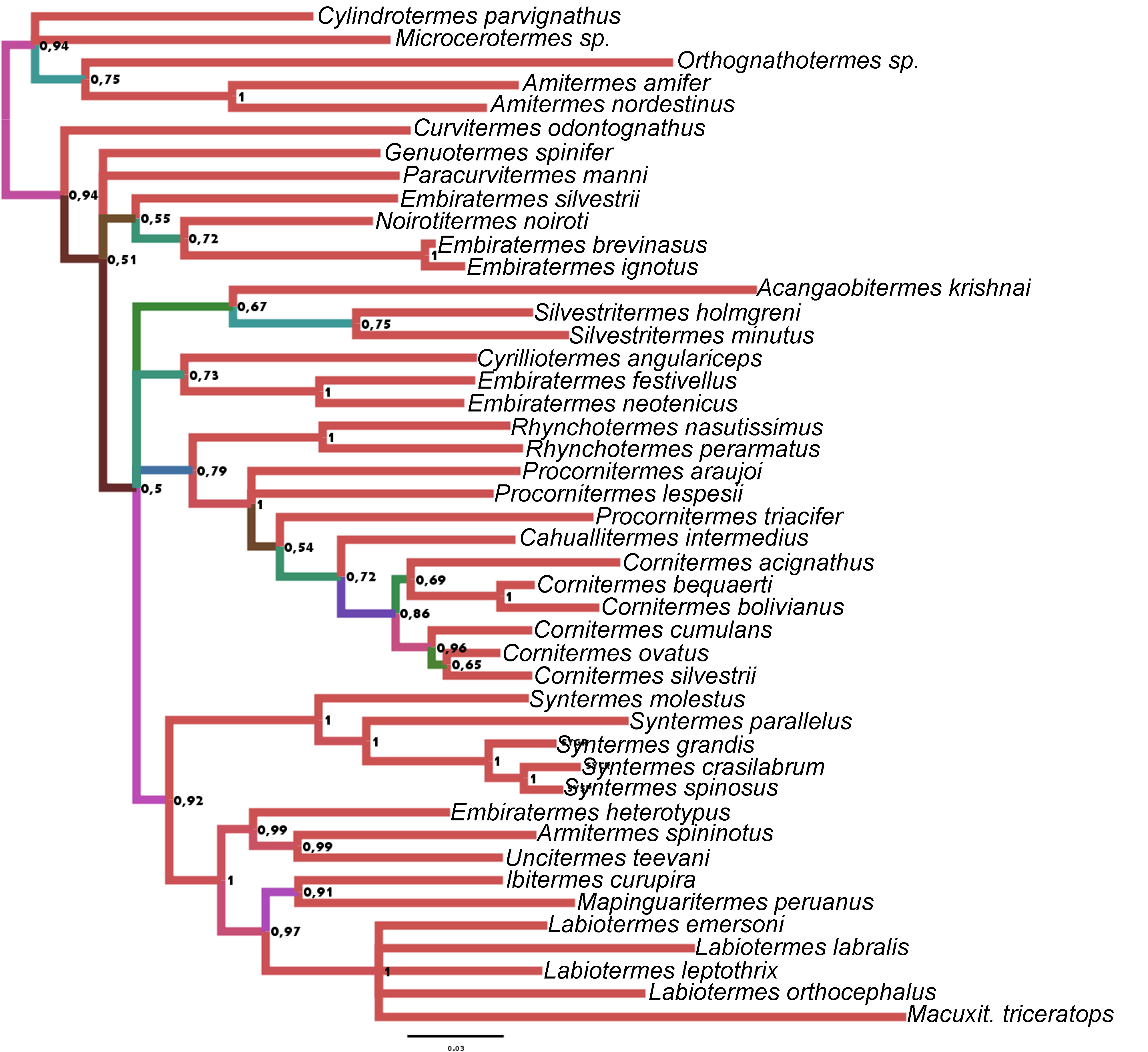

Supplement: S13 Fig — The respective posterior probability is indicated above each node, the branch color represents the posterior probability. (TIF) [file pone.0174366.s014.tif]

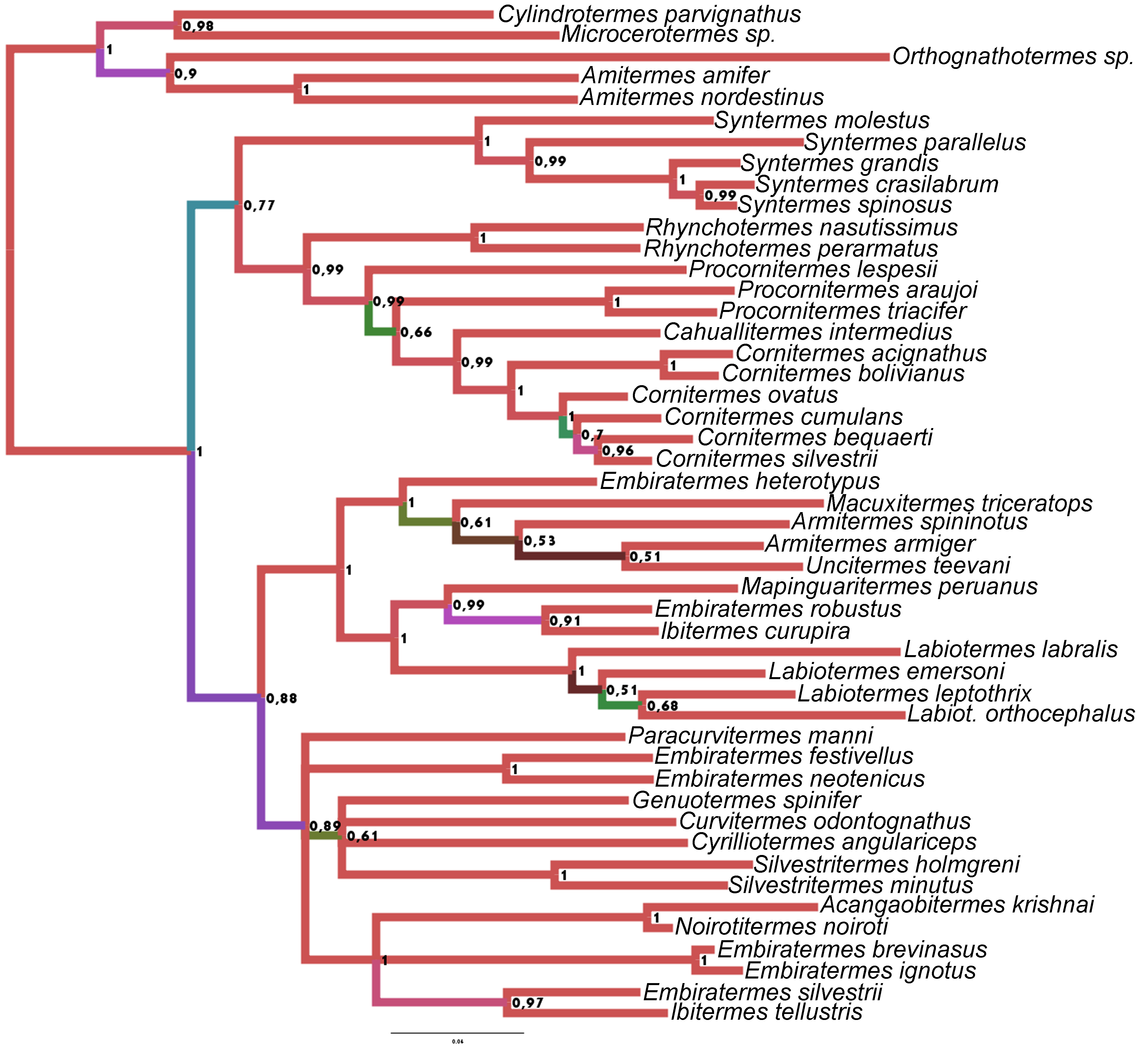

Supplement: S14 Fig — The respective posterior probability is indicated above each node, the branch color represents the posterior probability. (TIF) [file pone.0174366.s015.tif]

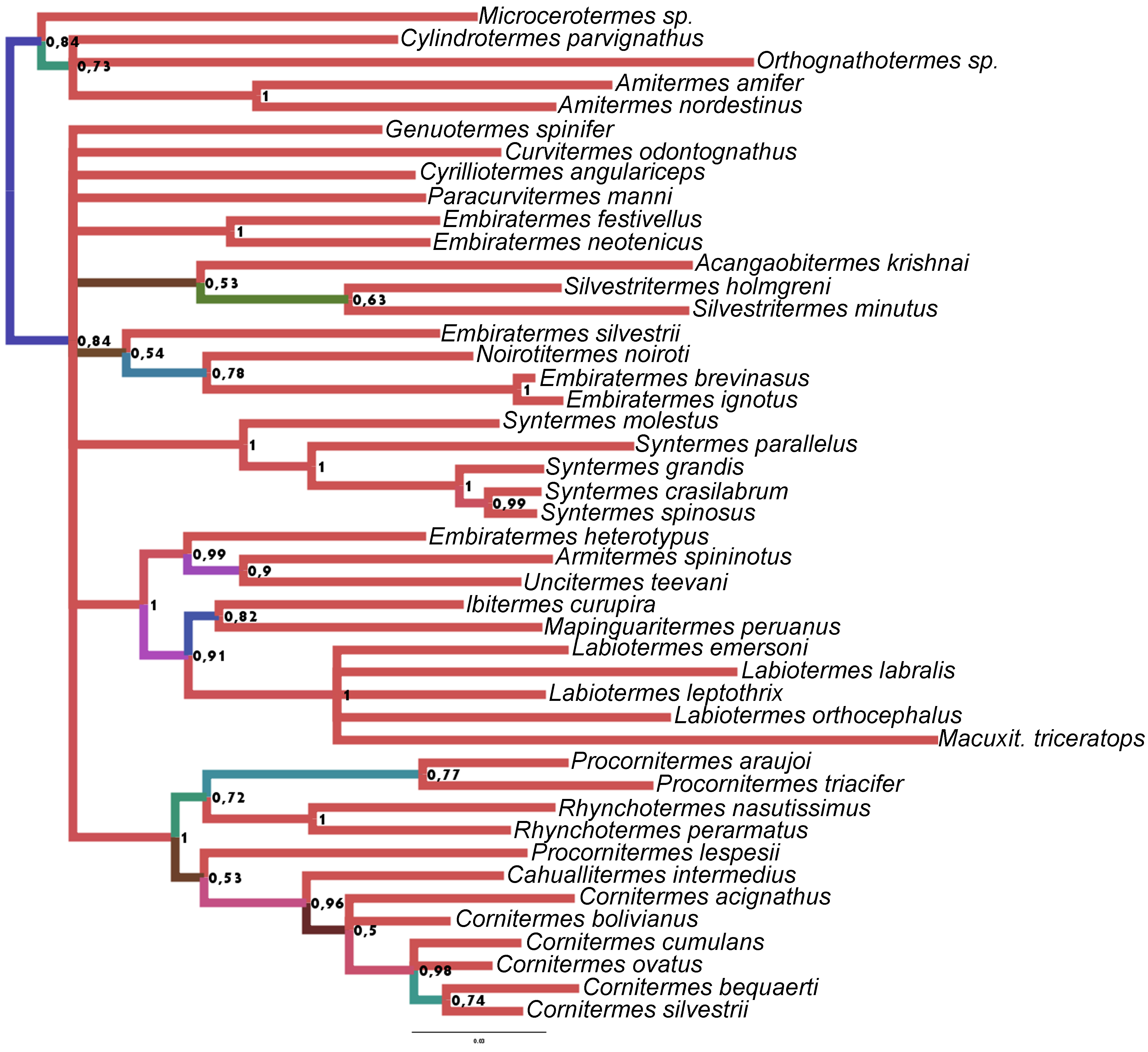

Supplement: S15 Fig — The respective posterior probability is indicated above each node, the branch color represents the posterior probability. (TIF) [file pone.0174366.s016.tif]

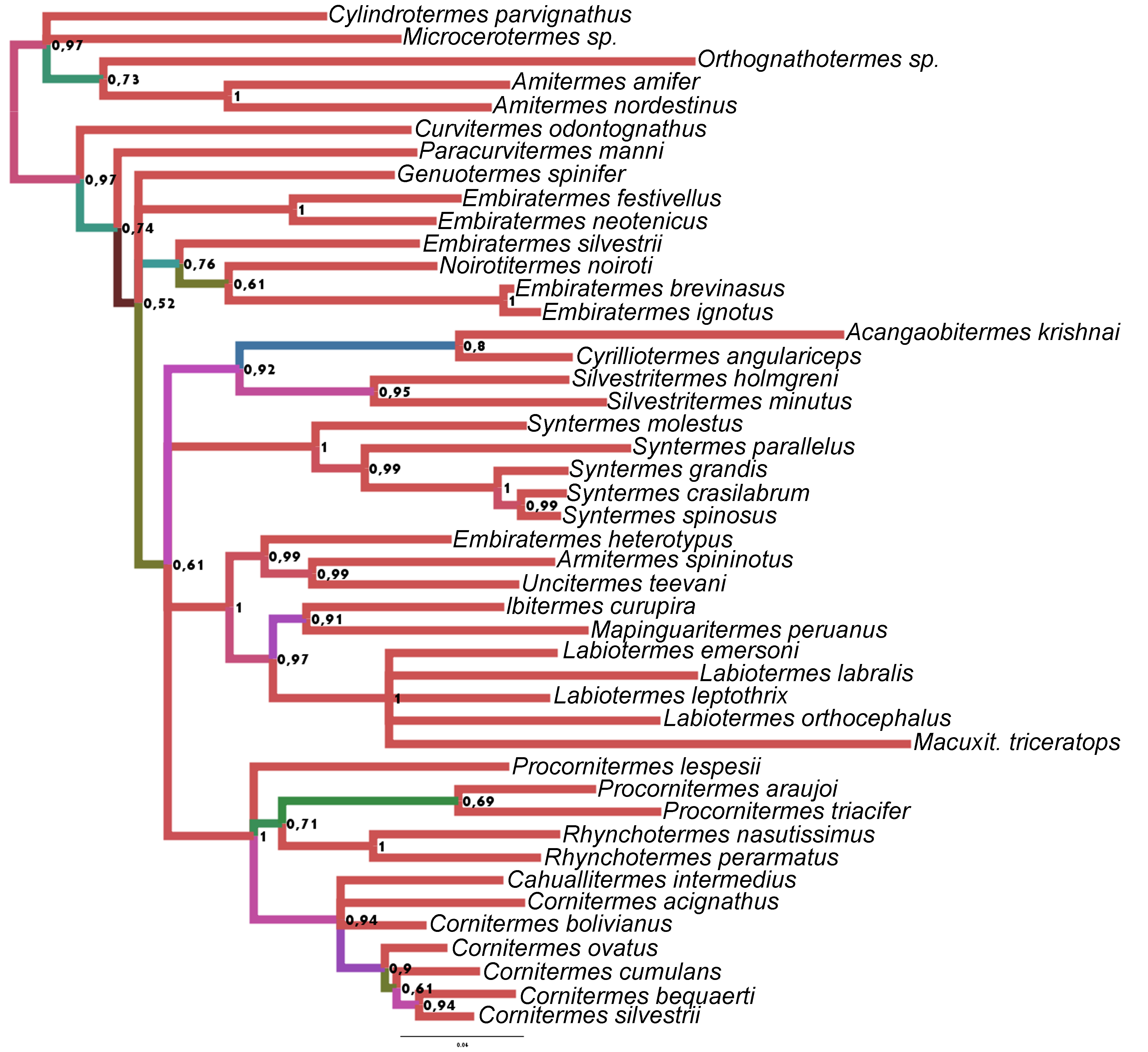

Supplement: S16 Fig — The respective posterior probability is indicated above each node, the branch color represents the posterior probability. (TIF) [file pone.0174366.s017.tif]
