# Supplementary material for: Phylogenetic reconstruction of Syntermitinae (Isoptera, Termitidae) based on morphological and molecular data
Source: PLoS One. 2017 Mar 22;12(3):e0174366. doi: 10.1371/journal.pone.0174366 (PMC5362239; doi:10.1371/journal.pone.0174366)
Supplement: S1 File — (DOCX) [file pone.0174366.s018.docx]

Information about the material used for DNA extractions in this work.

For each specimens used for DNA extraction there a testimonial material, composed by remain colony individuals, which are formally deposited in the MZUSP public depository (indicated by the lot number).

UHE = hydroelectric empowerment, P.N = National park.

|  | MZUSP Lot number | Place of collection, Municipality/State or province acronym, Country, ( Place of collection coordinates), date of collection, collector names. |
| --- | --- | --- |
| Termitinae |  |  |
| *Amitermes amifer* | 23727 | UHE Santo Antônio (Módulo de Jaci Paraná), Porto Velho/RO, Brasil, (Lat. -9.453585; Long. -64.39452), 6.iii.2012, R. Santos & J. Cabral col. |
| *Amitermes nordestinus* | 16373 | Poço Redondo/SE, 20.iv.2012, A.B.Viana col. |
| *Cylindrotermes parvignathus* | 23881 | Reserva Jataí, Luiz Antonio/SP, Brasil, 19.vi.2013, E.M.Cancello col. |
| *Genuotermes spinifer* | 16354 | UHE Santo Antônio (Módulo de Jirau), Porto Velho/RO, Brasil, (Lat. -9,3115; Long. -64,726), 18.ix.2010, T.F.Carrijo & J.Cabral col. |
| *Microcerotermes* sp. | 21513 | UHE Santo Antônio, Distrito de Jaci Paraná, módulo de Ilha da Pedra, Porto Velho/RO, Brasil, (Lat. -9,169969; Long. -64,616611), 21.xi.2011, M.M.Rocha & J.Cabral col. |
| *Orthognathotermes* sp. | 16233 | P.N. Serra da Canastra (Lat. -20.225; Long. -46.558)/MG, Brasil, 17.x.2012, M.M.Rocha col. |
| Syntermitinae |  |  |
| *Acangaobitermes krishnai* | 13670 | Módulo de Teotônio, Porto Velho/RO, Brasil, 29.ix.2010, T.F.Carrijo & R.G.Santos col. |
| *Armitermes spininotus* | 24420 | P.N. Grande Sertão Veredas/MG, Brasil, 12.x.2012, R.G.Santos col. |
| *Cahuallitermes intermedius* | 15463 | 17,5 Km E. of Tizimin, Iucatã, México, (Lat. 21.161, Long. -87.992), 07.xii.1997, Chase & Mangold col. |
| *Cornitermes acignathus* | 24421 | P.N. Serraria Yariguies , Colômbia , 2012, A.P.Vargas col. |
| *Cornitermes bequaerti* | 15970 | Sítio Areia de Minas, Arceburgo/MG, Brasil, vi.2012, E.M.Cancello col. |
| *Cornitermes bolivianus* | 20596 | UHE Santo Antônio, Distrito de Jaci Paraná (módulo de Ilha da Pedra), Porto Velho/RO (Lat. -9,154393; Long. -64,626076), 01/06/2012, M.M.Rocha & J.Cabral col. |
| *Cornitermes cumulans* | 24423 | Faz. Garça Branca, São Roque de Minas/ MG, Brasil, 2.iv.2012, J.P. Constantini & T.F. Carrijo col. |
| *Cornitermes ovatus* | 20617 | UHE Santo Antônio, Distrito de Jaci Paraná (módulo de Três Praias), Porto Velho/RO (Lat. -9.45022; Long. -64.36745), 24/11/2011, M.M.Rocha & J.Cabral col. |
| *Cornitermes silvestrii* | 16232 | P.N. Grande Sertão Veredas/MG, Brasil, (Lat. -15,413; Long. -45,917), 12.x.2012, M.M. Rocha col. |
| *Curvitermes odontognathus* | 20705 | UHE Santo Antônio, Distrito de Jaci Paraná (módulo de Morrinhos), Porto Velho (Lat. -9,029384 Long. -64,249984), 02/09/2011, R.G.Santos & J.Cabral col. |
| *Cyrilliotermes angulariceps* | 20709 | UHE de Jirau, Distrito de Mutum-Paraná (módulo de Mutum), Porto Velho/RO (Lat. -9.572735; Long. -65.061017), 31/03/2012, M.M.Rocha & R.G.Santos col. |
| *Embiratermes brevinasus* | 24424 | L5 Football Field, Cayenne, French Guyane, (5° 04.44 N, 53° 03.46 W), 2013,Y. Sbotnick col. |
| *Embiratermes festivellus* | 24425 | Sítio Areia de Minas, Arceburgo/MG, Brasil, vi.2012, E.M.Cancello col. |
| *Embiratermes heteropterus* | 24427 | Sítio Areia de Minas, Arceburgo/MG, Brasil, 5.viii.2012, J. L.Figueiredo |
| *Embiratermes ignotus* | 20810 | UHE de Santo Antônio, Distrito de Jaci Paraná (módulo de Três Praias), Porto Velho/RO (Lat. -9,451217; Long.-64,376033), 06/03/2012, T.F.Carrijo & J.Cabral col. |
| *Embiratermes neotenicus* | 23830 | UHE de Jirau, Porto Velho/RO, 18.ii.2013, M.Ulysséa & A. Barbão col. |
| *Embiratermes silvestrii* | 24428 | P.N. Grande Sertão Veredas/MG, Brasil, 15.x.2012, T.F.Carrijo col. |
| *Ibitermes curupira* | 24429 | Mata do Buraquinho, João Pessoa/PB, Brasil, 16.xi.2012, F.M.S. Moura col. |
| *Labiotermes emersoni* | 16219 | P.N. Grande Sertão Veredas/MG (Lat. -15.424; Long. -45.886), 14.x.2012, M.M.Rocha col. |
| *Labiotermes labralis* | 14771 | UHE de Jirau, Distrito de Abunã, Porto Velho/RO (Lat. -9,596509; Long. -65,337133), 27/06/2011, S.P.Rosa & G.R.Mazão col. |
| *Labiotermes leptothrix* | 20997 | UHE de Santo Antônio, Distrito de Jaci Paraná (Módulo de Três Praias), Porto Velho/RO (Lat. -9,452049; Long. -64,385079), 24/11/2011, M.M.Rocha & J.Cabral col. |
| *Labiotermes orthocephalus* | 14829 | UHE de Jirau, Distrito de Abunã, Porto Velho/RO (Lat. -9,632081; Long. -65,438702), 26.vi.2011, S.P.Rosa & G.R.Mazão col |
| *Macuxitermes triceratops* | 16103 | R. B. Ilha de Maracá, Amajari /RR, Brasil, (3° 22’ 40” N; 61° 28’ 57” W), 1–17ix.2012, C.S.Dambros col. |
| *Mapinguaritermes peruanus* | 14490 | UHE de Jirau, Distrito de Mutum-Paraná (módulo de Mutum), Porto Velho/RO (Lat. -9,606903; Long. -65,045762), 10/01/2011, M.M.Rocha & L.P.Prado col. |
| *Noirotitermes noiroti* | 24430 | P.N. Grande Sertão Veredas (Lat. -15.424; Long. -45.886)/MG, 14.x.2012, M.M.Rocha col. |
| *Paracurvitermes manni* | 21026 | UHE Santo Antônio/RO (Lat. -8.838231; Long. -64.065542), 9.vi.2012, M.M.Rocha & J. Cabral col. |
| *Procornitermes araujoi* | 16315 | P.N. Serra da Canastra (Lat. -20.308; Long. -46.525)/MG, 20.x.2012, M.M.Rocha col. |
| *Procornitermes lespesii* | 24431 | Parque do Ipiranga, São Paulo/SP, 18.i.2013, M.M.Rocha col. |
| *Procornitermes triacifer* | 24432 | Passo do Lontra, Base de Estudos do Pantanal da UFMS, Corumbá/MS,19.x.2012. T.F.Carrijo col. |
| *Rhynchotermes nasutissimus* | 15981 | Sítio Areia de Minas, Arceburgo/MG, 5.viii.2012, E.M.Cancello col. |
| *Rhynchotermes perarmatus* | 24433 | RNP Burbayar, Província do Panamá (9° 19’ 57” N; 78° 59’ 15” W), Panamá, 18–20.i.2013, R. Pinto |
| *Silvestritermes holmgreni* | 20549 | UHE de Santo Antônio, Distrito de Teotônio, Porto Velho/RO (Lat. -8,838231; Long. -64,065542), 09/06/2012, M.M.Rocha & J.Cabral col. |
| *Silvestritermes minutus* | 20553 | UHE de Jirau, Distrito de Mutum-Paraná (módulo de Caiçara), Porto Velho/RO (Lat. -9,451345; Long. -64,843903), 30/03/2012, M.M.Rocha & R.G.Santos col. |
| *Syntermes crassilabrum* | 21044 | UHE de Jirau, Distrito de Mutum-Paraná (módulo de Mutum), Porto Velho/RO (Lat. -9,588455; Long. -65,066459), 15/09/2011, T.F.Carrijo & L.R.Fernandes col. |
| *Syntermes grandis* | 16338 | P.N. Grande Sertão Veredas (Lat. -15.173; Long. -45.720)/MG, 11.x.2012, M.M.Rocha col. |
| *Syntermes molestus* | 21069 | UHE de Jirau, Distrito de Mutum-Paraná (módulo de Mutum), Porto Velho/RO (Lat. -9,574046; Long -65,071677), 23/06/2012, R.G.Santos & K.Kawamishi col. |
| *Syntermes parallelus* | 14753 | UHE de Santo Antônio, Distrito de Jaci Paraná (módulo de Três Praias), Porto Velho/RO (Lat. -9,4491, Long. -64,357719), 20/09/2010, T.F.Carrijo & R.G.Santos col. |
| *Syntermes spinosus* | 21155 | UHE de Jirau, Distrito de Abunã, Porto Velho/RO (Lat. -9,619116; Long. -65,460881), 12/01/2012, R.G.Santos & J.P.Constantini col. |
| *Uncitermes teevani* | 20574 | UHE de Jirau, Distrito de Mutum-Paraná (módulo de Mutum), Porto Velho/RO (Lat. -9,591526, Long. -65,05023), 10/01/2012, R.G.Santos & J.P.Constantini col. |
